# Modeling of Temporal Exposure to the Ambient Environment and Eczema Severity

**DOI:** 10.1016/j.xjidi.2021.100062

**Published:** 2021-10-09

**Authors:** Bjorn R. Thomas, Xiang L. Tan, Shagayegh Javadzadeh, Elizabeth J. Robinson, Bryan S. McDonald, Malvina A. Krupiczojc, Syedia R. Rahman, Samiha Rahman, Rehana A. Ahmed, Rubina Begum, Habiba Khanam, David P. Kelsell, Jonathan Grigg, Robert J. Knell, Edel A. O’Toole

**Affiliations:** 1 Centre for Cell Biology and Cutaneous Research, Blizard Institute, Barts and the London School of Medicine and Dentistry, Queen Mary University of London, London, United Kingdom; 2 Department of Dermatology, The Royal London Hospital, Barts Health NHS Trust, London, United Kingdom; 3 School of Biological and Behavioural Sciences, Queen Mary University of London, London, United Kingdom

**Keywords:** AIC, Akaike Information Criterion, EASI, Eczema Area and Severity Index, EseC, European Socio-Economic Classification, GAM, generalized additive model, IGA, Investigators Global Assessment, MAv, moving average, NO, nitric oxide, NO_2_, nitrogen dioxide, NO_x_, nitrogen oxide, O_3_, ozone, PM, particulate matter, SCORAD, SCORing Atopic Dermatitis, SE, standard error, THEA, Tower Hamlets Eczema Assessment, VOC, volatile organic compound

## Abstract

Atopic eczema is a common and complex disease. Missing genetic hereditability and increasing prevalence in industrializing nations point toward an environmental driver. We investigated the temporal association of weather and pollution parameters with eczema severity. This cross-sectional clinical study was performed between May 2018 and March 2020 and is part of the Tower Hamlets Eczema Assessment. All participants had a diagnosis of eczema, lived in East London, were of Bangladeshi ethnicity, and were aged <31 years. The primary outcome was the probability of having an Eczema Area and Severity Index score > 10 after previous ambient exposure to commonly studied meteorological variables and pollutants. There were 430 participants in the groups with Eczema Area and Severity Index ≤ 10 and 149 in those with Eczema Area and Severity Index > 10. Using logistic generalized additive models and a model selection process, we found that tropospheric ozone averaged over the preceding 270 days was strongly associated with eczema severity alongside the exposure to fine particles with diameters of 2.5 μm or less (fine particulate matter) averaged over the preceding 120 days. In our models and analyses, fine particulate matter appeared to largely act in a supporting role to ozone. We show that long-term exposure to ground-level ozone at high levels has the strongest association with eczema severity.

## Introduction

Eczema is the most common inflammatory skin disease (Karimkhani et al., 2017). Large population studies have shown that the prevalence of eczema is rising in developing countries as they industrialize, suggesting an environmental effect (Odhiambo et al., 2009; Williams et al., 2008).

Meteorological factors and ambient air pollution have been reported to be associated with the severity and prevalence of eczema (Ahn, 2014; Kathuria and Silverberg, 2016; Kim et al., 2017; Langan et al., 2006; Silverberg et al., 2013). Studies have shown that fine particles with diameters of 10 μm or less (particulate matter [PM]_10_) are associated with a decrease in prevalence (Kathuria and Silverberg, 2016), whereas others have shown these to be associated with an increase in disease incidence (Belugina et al., 2018).

With respect to pollution, it has become clear that fine particles with diameters of 2.5 μm or less (PM_2.5_) and tropospheric (ground-level) ozone (O_3_) cause the majority of mortality and disease related to pollution (GBD 2013 Risk Factors Collaborators et al., 2015; Lim et al., 2012). PM_2.5_ is not a single pollutant but a mixture of many chemical species, including black carbon emitted directly from fuel combustion. Combustion of fossil fuels contributes a large amount to PM_2.5_ concentrations (Chow and Watson, 2002). A complicating feature when studying the effect of pollution on disease is that the composition of pollutants varies between countries and cities, particularly PM_2.5_, making comparisons difficult (Li et al., 2019b).

Ground-level O_3_ is created by photolytic reactions between volatile organic compounds (VOCs) and nitrogen oxides (NO_x_), mainly nitric oxide (NO) and nitrogen dioxide (NO_2_). In a system without VOCs, NO_2_ and oxygen combine to create O_3_ and NO. O_3_ in turn reacts with NO to recreate NO_2_ in a balanced cycle with no net increase in O_3_ levels (Zhang et al., 2019). This balanced cycle depends on stable solar intensity, temperature, and a constant ratio of available NO_2_ and NO. In reality, the situation is more complicated because VOCs continuously arise from things such as personal care products, pesticides (McDonald et al., 2018), and even isoprene released from trees (Fehsenfeld et al., 1992). These compounds can form free radicals, which compete with O_3_ in its reaction with NO, resulting in increasing O_3_ levels. Increasing worldwide temperatures and escalating fuel combustion favor NO_x_ formation, which also results in surging O_3_ production. Interestingly, in urban settings, falling NO_x_ levels lead to an elevation in O_3_ owing to repartitioning of the NO/NO_2_/O_3_ balance (Lee et al., 2020). This was seen during the March 2020 COVID-19 lockdown in London, United Kingdom. The increase in O_3_ levels seen during this period was compounded by higher UV levels, temperatures, and biogenic VOC production from plants in Southern England (Fitzky et al., 2019; Lee et al., 2020). As we move away from NO_x_ emissions, we can expect higher O_3_ levels.

It is also known that PM_2.5_ can act as a sink for the free radicals needed for O_3_ formation. When PM concentrations fall, urban O_3_ levels also increase (Li et al., 2019a; Ma et al., 2016), illustrated by events in China where active efforts were implemented to reduce annual PM_2.5_ and NO_x_ levels. This has been associated with rising O_3_ concentrations over the last few years (Li et al., 2019a; Ma et al., 2016). It is thought that controlling man-made VOCs will help combat this paradoxical O_3_ increase (Le et al., 2020; Lee et al., 2020). Interestingly, wind also plays an important role in this system. Despite pollution control measures in one region, pollution, including O_3_ from adjacent upwind areas, can still lead to increased mortality rates in the pollution-controlled downwind areas (Dedoussi et al., 2020).

Data exist to support the role of pollutants in the pathogenesis of eczema (Hendricks et al., 2020). In mouse models, diesel exhaust particles drive eczema by activating the aryl hydrocarbon receptor (Hidaka et al., 2017). The aryl hydrocarbon receptor also acts as an O_3_ sensor in the skin (Afaq et al., 2009). Activation of the aryl hydrocarbon receptor can induce proinflammatory COX-2 (Lee et al., 2016) and IL-8 (Tsuji et al., 2011) and induce the expression of TRPA1 and TRPV1 in primary afferent nerve channels causing itch (Elitt et al., 2006; Hidaka et al., 2017). O_3_ exposure led to the activation of the Jak2/signal transducer and activator of transcription 3 and Akt1/NF-κB pathways in mouse lungs (Mishra et al., 2016). In the skin, these pathways are relevant to eczema pathogenesis (Howell et al., 2019; Rogerson and O’Shaughnessy, 2018), with the Jak/signal transducer and activator of transcription pathway being an important therapeutic target.

The temporal association between individual pollutants, meteorological variables, and eczema is also not fully understood. In this cross-sectional clinical study, we investigated the association of these factors with eczema severity using a cohort of 579 children and young adults of Bangladeshi ethnicity with eczema (Table 1) in East London, United Kingdom. We have previously shown that this population has an increase in variation of the *FLG* gene (Pigors et al., 2018). We investigated the exposure period and value of a pollutant or meteorological variable that has the strongest association with eczema severity. Using logistic generalized additive models (GAMs) and a model selection approach (explained in Materials and Methods), we investigated which of the meteorological and pollutant variables were most influential in the models of eczema severity.

**Table 1:** Demographics and Clinical Characteristics of THEA Cohort

	EASI ≤ 10 Cohort (n = 430)	EASI > 10 Cohort (n = 149)
Characteristic		
Age		
Median (Q1–Q3)	9.25 (4.70–14.50)	11.60 (6.80–16.50)
Sex		
Female (%)	193 (44.88)	53 (35.57)
Male (%)	237 (55.12)	96 (64.42)
BMI		
Median (Q1–Q3)	17.79 (15.75–21.86)	18.63 (15.72–23.66)
ESEC		
Class 1-3: Higher (%)	141 (32.79)	31 (20.81)
Class 4-6: Middle (%)	71 (16.51)	45 (30.20)
Class 7-9: Working(%)	218 (50.70)	73 (48.99)
EASI		
Median (Q1–Q3)	2.80 (1.13–5.4)	17.95 (13.20–27.60)
TEWL		
Median (Q1–Q3)	10.82 (8.84–13.63)	14.50 (11.53–20.00)
SH		
Median (Q1–Q3)	26.53 (21.33–32.71)	21.33 (14.87–27.10)
Season		
Spring (%)	87 (20.23)	18 (12.08)
Summer (%)	138 (32.09)	46 (30.87)
Autumn (%)	128 (29.77)	46 (30.87)
Winter (%)	77 (17.91)	39 (26.17)
Investigator		
Research nurse (%)	252 (58.60)	36 (24.16)
Clinical fellow (%)	139 (32.33)	95 (63.76)
Consultant (%)	15 (3.49)	6 (4.03)
Other trained (%)	24 (5.58)	12 (8.05)

Abbreviations: BMI, body mass index; EASI, Eczema Area and Severity Index; EASI10, EASI score ≤ or > 10; ESEC, European Socio-economic Classification; n, number or participants; Q1–Q3, 25th and 75th percentiles; SH, skin hydration measured using a corneometer; TEWL, trans-epidermal water loss measured with a Tewameter.

## Results

### Participants

A total of 579 participants were included in the analysis, with 430 participants in the group with Eczema Area and Severity Index (EASI) ≤ 10 and 149 in the group with EASI > 10 (Table 1).

### Models: individual environmental factors

**Figure 1:**
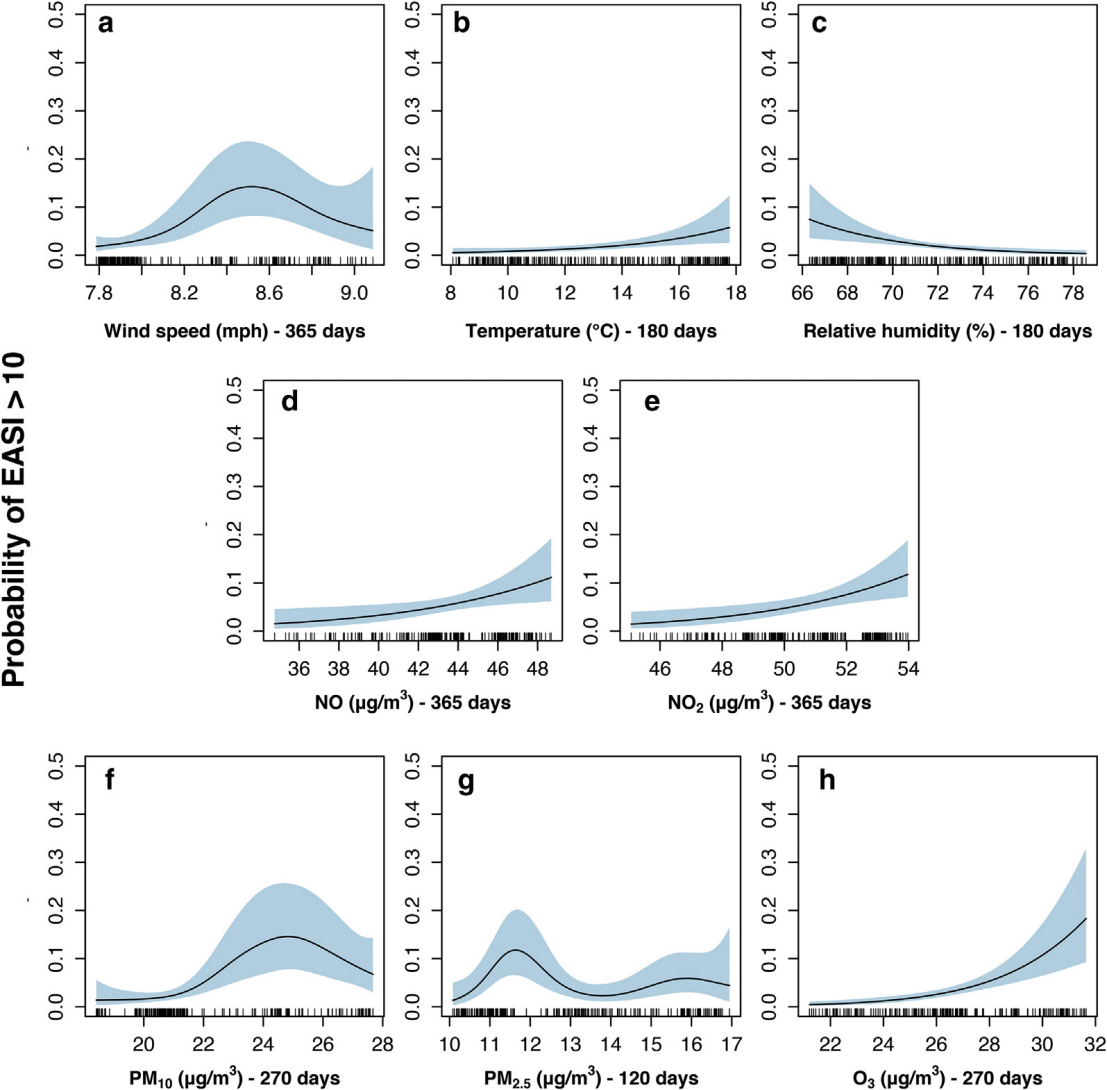
**Target variable partial effect plots from the best supported models selected from the 10 model variable set using Δ6 AIC.** Probability of having an EASI > 10 (y-axis) versus the mean ambient weather and pollution variables (x-axis): (**a**) Wind 365, Wind speed averaged over 365 days (**b**) Temperature 180, Temperature averaged over 180 days (**c**) Hum 180, Relative humidity averaged over 180 days (**d**) NO 365 , NO averaged over 365 days (**e**) NO_2_ 365, NO_2_ averaged over 365 days (**f**) PM_10_ 270 , PM_10_ over 270 days (**g**) PM_2.5_ 120, PM_2.5_ averaged over 120 days (**h**) O_3_ 270, O_3_ averaged over 270 days. The light blue shaded area represent the 95% confidence intervals. The small dashes overlying the x-axis are known as the rug and they represent all of the cases. Note: Inability to draw a horizontal line throughout the 95% confidence interval indicates the smooth is significant. AIC, Akaike Information Criterion; EASI, Eczema Area and Service Index; NO, nitric oxide; NO_2_, nitrogen dioxide; O_3_, ozone; PM_2.5_, 2.5 μm particulate matter; PM_10_, 10μm particulate matter.

**Figure 2:**
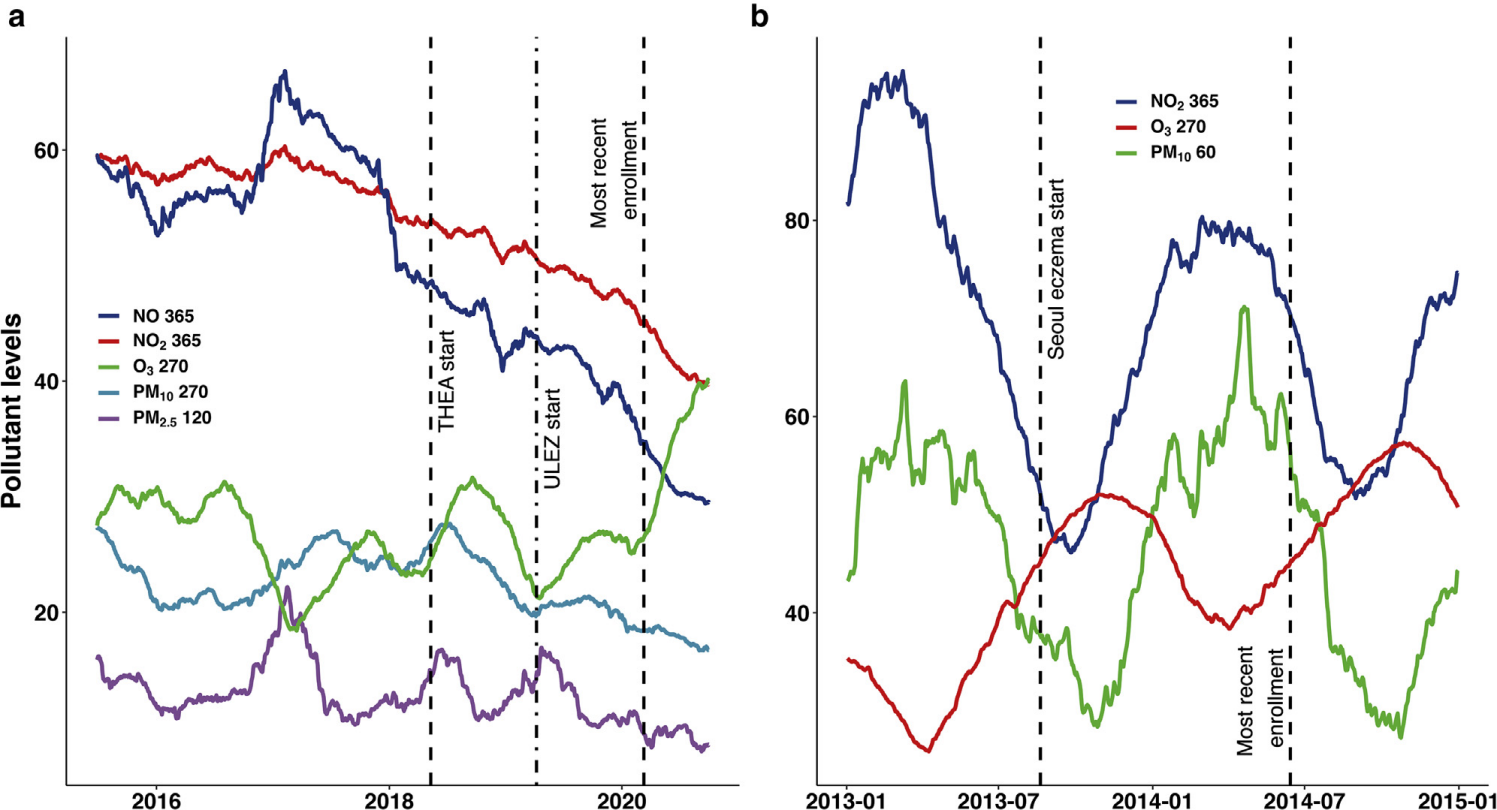
**Main pollutant levels during the entirety of the THEA study (a) and Korean dataset (b).** (**a**) Moving average line graphs for selected single pollutant models from 28 June 2015 to 30 September 2020. Dashed lines represents the recruitment start date and most recent enrolment. The dash–dot line represents the date in which the ULEZ was introduced in central London. NO 365 – NO levels over the last 365 days; NO_2_ 365, NO_2_ over the last 365 days; O_3_ 270, Ground level O_3_ over the last 270 days; PM_10_ 270, average level of PM_10_ over the last 270 days; PM_2.5_*,* 2.5μm particulate matter; PM_2.5_ 120, average level of PM_2.5_ over the last 120 days. (**b**) Three available pollutants from Daechi-dong, Gangnam-gu, Seoul, Korea from January 2013 to January 2015. NO_2_ 365, NO_2_ over the last 365 days; O_3_ 270, Ground level O_3_ over the last 270 days; PM_10_ 60, average level of PM_10_ over the last 60 days. ∗All values in μg/m^3^. NO, nitric oxide; NO_2_, nitrogen dioxide; O_3_, ozone; PM_2.5_, 2.5 μm particulate matter; PM_10_ , 10μm particulate matter; THEA, Tower Hamlets Eczema Assessment; ULEZ, Ultra Low Emission Zone.

A total of 10 models per environmental factor were run to examine the association of each variable with the severity of eczema. The 10 models include variables matched to the day of participant recruitment and nine increasing moving averages (MAvs) preceding recruitment (Figure 1). For each variable, the MAv threshold model with the lowest Akaike Information Criterion (AIC) was chosen as the top-performing model (Supplementary Tables S1 and 2) (Burnham and Anderson, 2004, Burnham and Anderson, 2002). The range in values of the chosen variables over the entirety of the study is shown in Figure 2 and Supplementary Table S3.

The partial effects plots (Figure 1) show that increasing temperature and relative humidity averaged over 180 days were associated with a rise and fall in the probability of having an EASI > 10, respectively. In contrast, increasing wind speed averaged over 365 days and PM_10_ levels averaged over 270 days were associated with a higher probability of EASI > 10. It is interesting to note that the model that is best supported for PM_2.5_ (averaged over 120 days) appears to be mostly associated with an increased probability of EASI >10 at the lower ranges. For O_3_ levels (averaged over 270 days), there is an almost linear increase in the probability of EASI > 10 from 0 to about 20%.

Of these models, the O_3_ 270 (O_3_ averaged over 270 days) model, according to the AIC, performed best. The PM_10_ 270 (PM_10_ levels averaged over 270 days) and wind 365 (wind speed averaged over 365 days) were the second and third best-performing models, respectively, with significantly less support (ΔAIC > 7 from the O_3_ 270 model). All other environmental parameters individually modeled have ΔAIC > 10, indicating very little support when compared with O_3_ 270.

### Top model selection

All environmental variables were included together in a base model to understand which variables associated best with EASI 10 in a more comprehensive system. Backward selection using ΔAIC of 6 and the nesting rule was used to remove variables with less support, leaving nine models in the top set (Table 2 and Supplementary Figure S5a‒h and Supplementary Table S4a‒h).

**Table 2:** Top Model Set

Model Number	Equation	*R*^2^_Adj_	AIC		C-Index^1^ (95% CI)
**Base Model Prior to Backward Selection and Double Penalty Selection**					
Base	EASI10 ∼ NO 365/NO_2_ 365 Dim1 + PM_10_ 270/Wind 365 Dim 1 + Temp 180/Hum 180 Dim 1 + PM_2.5_ 120 + O_3_ 270 + SH + TEWL + Age + Lon:Lat + BMI + Season + Sex + Investigator + EseC class	0.365	485.16		0.87 (0.83–0.91)
**Nine Best Supported Models Backward Selection (Δ6 AIC and Nesting Rule)**				**ΔAIC**	
**1** (4a)	EASI10 ∼ PM_2.5_ 120 + O_3_ 270 + SH + TEWL + Age + BMI + Season + Sex + Investigator + EseC class	0.368	478.17	0	0.87 (0.83–0.91)
**2** (5f)	EASI10 ∼ PM_2.5_ 120 + O_3_ 270 + SH + TEWL + Age + Season + Sex + Investigator + EseC class	0.365	478.73	0.56	0.86 (0.82–0.90)
**3** (5h)	EASI10 ∼ PM_2.5_ 120 + O_3_ 270 + SH + TEWL + Age + BMI + Season + EseC class	0.360	479.56	1.39	0.87 (0.83–0.97)
**4** (5a)	EASI10 ∼ O_3_ 270 + SH + TEWL + Age + BMI + Season + Sex + Investigator + EseC class	0.351	480.39	2.22	0.86 (0.82–0.90)
**5** (5g)	EASI10 ∼ PM_2.5_ 120 + O_3_ 270 + SH + TEWL + Age + BMI + Sex + Investigator + EseC class	0.353	480.93	2.76	0.86 (0.82–0.90)
**6** (4j)	EASI10 ∼ NO 365/NO_2_ 365 Dim1 + PM_2.5_ 120 + O_3_ 270 + SH + TEWL + Age + BMI + Season + Sex + Investigator + EseC class	0.363	482.53	4.36	0.87 (0.83–0.90)
**7** (5i)	EASI10 ∼ PM_2.5_ 120 + O_3_ 270 + SH + TEWL + Age + BMI + Season + Sex + Investigator + EseC class	0.363	482.55	4.38	0.86 (0.82–0.90)
**8** (5j)	EASI10 ∼ PM_2.5_ 120 + O_3_ 270 + SH + TEWL + Age + BMI + Season + Sex + Investigator	0.365	483.16	4.99	0.87 (0.83–0.91)
**9** (2e)	EASI10 ∼ NO 365/NO_2_ 365 Dim1 + PM_10_ 270/Wind 365 Dim 1 + Temp 180/Hum 180 Dim 1 + PM_2.5_ 120 + SH + TEWL + Age + BMI + Season + Sex + Investigator + EseC class	0.360	483.54	5.37	0.87 (0.83–0.91)

Abbreviations: AIC, Akaike Information Criterion; BMI, body mass index; CI, confidence interval; ESeC, European socio-econmic classification; Hum 180, average relative humidity over the last 180 days; Investigator, researcher who perform the EASI score per participant; Lon:Lat, longitude and latitude; NO_2_ 365, average nitrogen dioxide level over last 365 days; NO 365, average level nitric oxide over last 365 days; NO 365/NO_2_ 365 Dim 1 , Dimension 1 of PCA between NO 365 and NO_2_ 365; O_3_ 270, average level of ozone over the last 270 days; PM_2.5_ 120, average level of PM_2.5_ over the last 120 days; PM_10_ 270, average level of PM_10_ over the last 270 days; PM_10_ 270/Wind 365 Dim 1 , Dimension 1 of PCA between PM_10_ 270 and Wind 365; *R*^2^_Adj_, Adjusted R-squared; SH, skin hydration; Temp 180, average temperature over the last 180 days; TEWL, transepidermal water loss; Wind 365, average wind speed over last 365 days.

1 C-Index; Concordance statistic, measure of goodness of fit, equal to the area under the Receiver Operating Characteristic curve.

O_3_ 270 is included as a variable in eight of the nine models in the top set and is only missing from the one with the highest ΔAIC, so the inclusion of this variable is well-supported, in contrast with NO 365/NO_2_ 365, which is only in two of the top set models and therefore has less support. PM_2.5_ 120 is also present in eight of nine models, but the partial effects were significant only in models 5 and 6. Model 9, with the highest ΔAIC (5.37), contains all environmental variables except O_3_ 270.

**Figure 3:**
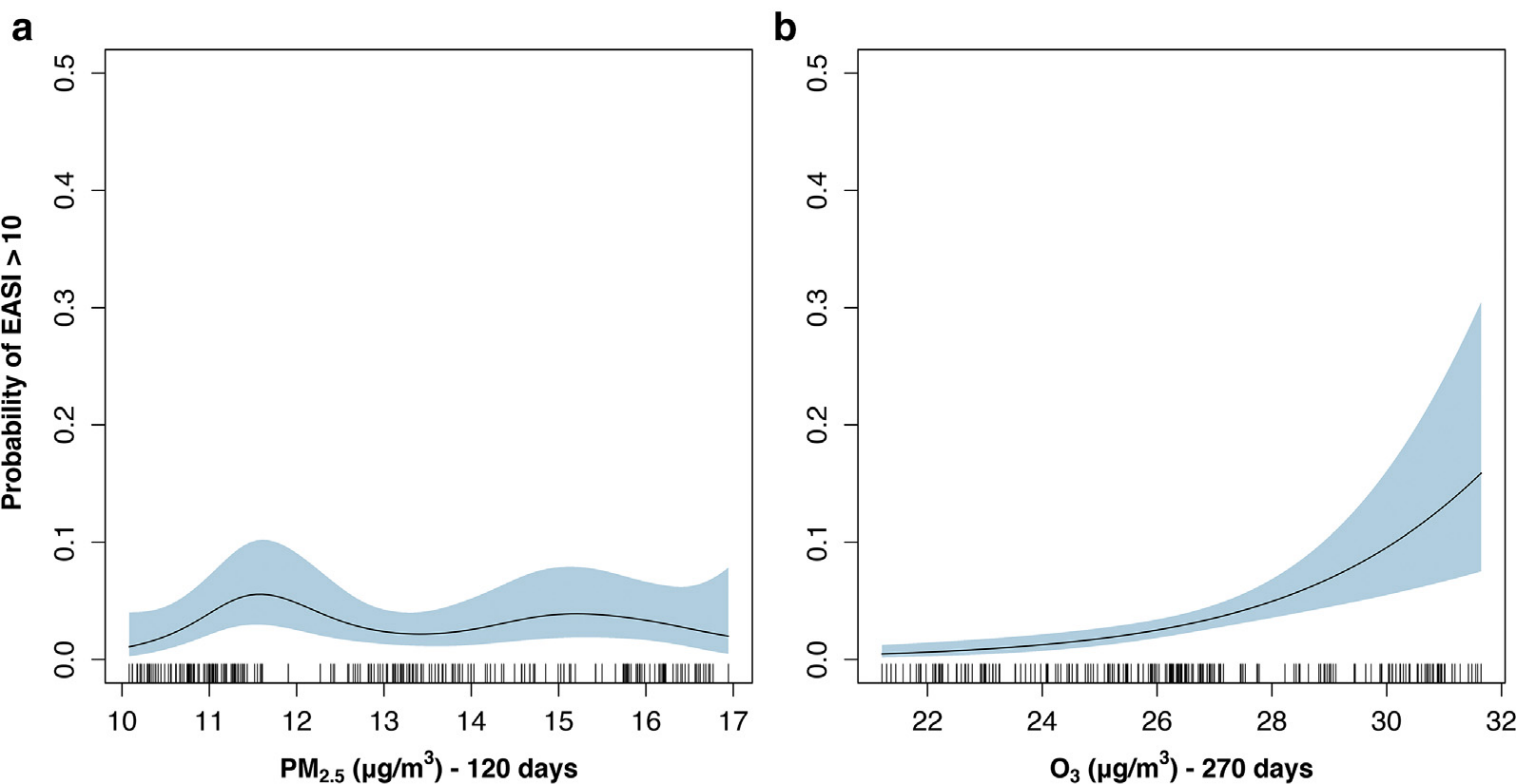
**Top performing model: Smooth terms (nonlinear parameters) partial effects plots: O_3_ and PM_2.5_ versus probability of EASI > 10.** PM_2.5_ and O_3_ partial effects plot from top performing model - using Δ6 AIC and nesting rule. (**a**) PM_2.5_ 120, PM_2.5_ averaged over 120 days (*P* = 0.126) (**b**) O_3_ 270 (*P* = <0.001), O_3_ averaged over 270 days. Other partial effects for this model and for other models in the top model set can be seen in the Supplementary materials. Probability of having a EASI > 10 (y-axis) versus the mean ambient weather and pollution variables (x-axis): O_3_ 270, O_3_ over 270 days. The light blue shaded area represent the 95% confidence interval. The small dashes overlying the x-axis are known as the rug and they represent all of the cases. Note: Inability to draw a horizontal line throughout the 95% confidence interval indicates the smooth is significant. In this case PM_2.5_ 120 is not significant. AIC, Akaike Information Criterion; EASI, Eczema Area and Service Index; O_3_, ozone; PM_2.5_, 2.5 μm particulate matter.

Model 1 performed best in the set, and the partial effects for this model can be seen in Figure 3 and model statistics in Table 3. This model had good discriminatory ability for EASI 10 (area under the receiver operating curve/C-Index: 0.87; 95% CI = 0.83‒0.91; *P* < 0.001) (Supplementary Figure S6). In this model, as O_3_ 270 increases, the probability of EASI >10 increases to above 15%. The nonsignificant PM_2.5_ partial effect in model 1 has a similar shape to the single PM_2.5_ model (Figure 1g), with a bump at lower levels. When we look at the partial effects in the top set, O_3_ is significant in all models in which it is contained, including model 1, with an almost linear increase across its range.

**Table 3:** Top Performing Model: Linear Parameter ORs Versus EASI > 10

		R^2^_Adj_	AIC
		**C-Index**^1^	
**Linear Parameters:**			
	**OR**	**95% CI**	***P*-Value**
Season: Autumn (Ref)	—	—	—
Season: Spring	4.32	0.98–19.08	0.053
Season: Summer	3.56	1.27–9.97	0.015
Season: Winter	4.17	1.54-11.31	0.005
Female (Ref)	—	—	—
Male	1.62	0.96–2.71	0.068
Investigator 1 (Ref)	—	—	—
Investigator 2	1.86	0.92–3.78	0.085
Investigator 3	0.28	0.06–1.28	0.100
Investigator 4	1.10	0.38–3.22	0.860
ESeC class 1–3 (Ref)	—	—	—
ESeC class 4–6	2.79	1.39–5.58	0.004
ESeC class 7–9	1.39	0.76–2.55	0.282

Abbreviations: 95% CI, 95% confidence interval; AIC, Akaike Information Criterion; ESeC, European Socio-economic Classification; *R*^2^_Adj_ , Adjusted *R*^2^; Ref, reference.

1 Model performance metrics: R^2^ Adj, 0.37; AIC, 478.17; C-Index, 0.871; C-Index; Concordance statistic, measure of goodness of fit, equal to the area under the Receiver Operating Characteristic curve.

As mentioned earlier, the NO/NO_2_ composite appears only twice in the nine model top set with insignificant partial effects. When modeled individually (Supplementary Table S1), they both have an ΔAIC > 10 when compared with the single O_3_ 270 model. This gives less support for NO_x_ playing a direct role in influencing EASI 10.

**Figure 4:**
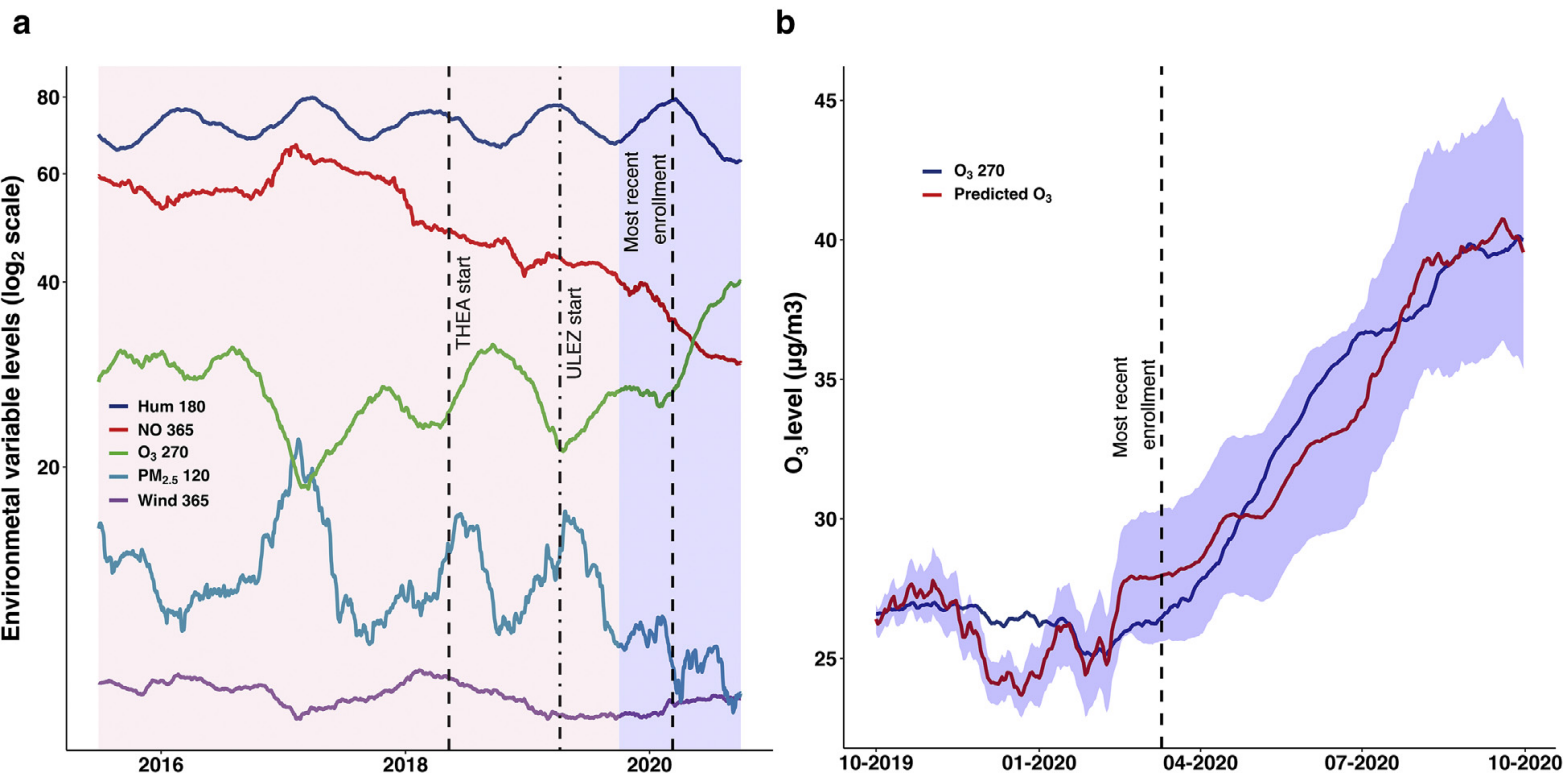
**Meteorological and pollutant levels for O_3_ 270 prediction.** Raw values for GAM training and O_3_ 270 prediction. (**a**) Environmental values found in Model 9 of the top model set in addition to O_3_ 270 from 28 June 2015 to 30 September 2020. The pink area represents the data range for the training set (28 June 2015 to 1 October 2019) to predict O_3_ 270 using NO 365, Hum 180, Wind 365 and PM_2.5_ 120 (adjusted R^2^ of 92.10). The blue area represents the test set (2 October 2019 to 30 September 2020) for O_3_ 270. (**b**) Predicted O_3_ 270 (red line) with 95% confidence interval in light blue from 2 October 2019 to 30 September 2020. Actual O_3_ 270 (dark blue) during that period. GAM, generalized additive model; Hum, humidity; NO, nitric oxide; O_3_, ozone; THEA, Tower Hamlets Eczema Assessment; ULEZ, Ultra Low Emission Zone.

Of note, model 9 with the highest ΔAIC did not contain O_3_ 270 but included dimension 1 of principal component analysis between (i) NO 365 and NO_2_ 365, (ii) PM_10_ 270 and wind 365, (iii) temperature 180 and humidity 180, and (iv) PM_2.5_ 120 alone. It is known that NO, wind speed, humidity, and PM_2.5_ can all affect O_3_ levels (Gorai et al., 2015; Kavassalis and Murphy, 2017; Li et al., 2019a). We therefore looked at the raw values for NO 365, wind 365, humidity 180, PM_2.5_ 120, and O_3_ 270 from June 28, 2015 to September 30, 2020. All variables appeared to be at least partially correlated–mostly inverse (Figure 4a). We trained GAM using default setting and no transformations on environmental data from 28 June 2015 to 1 October 2019 (training set) to see whether changes in NO 365, wind 365, humidity 180, and PM_2.5_ 120 account for changes in the levels of O_3_ 270 seen over that period. We then used the trained model (adjusted R_2_ of 92.10) to predict O_3_ 270 from 2 October 2019 to 30 September 2020 (test set). Despite the fact that the test data contained values of NO and PM_2.5_ not seen before in the training data, we were able to reasonably predict the dramatic O_3_ 270 spike during the Spring 2020 United Kingdom COVID-19 lockdown (Figure 4a). This experiment suggests that in general, changes in NO 365, wind 365, humidity 170, and PM_2.5_ 120 account for O_3_ 270 levels, therefore explaining the presence of model 9 in the top set. Rather than having individual direct effects on eczema severity (which they still may have), the environmental variables in model 9 may be acting as a surrogate for O_3_ 270.

In summary, a total of eight models directly include O_3_ 270 and PM_2.5_, with the ninth (model 9) possibly acting as a surrogate for O_3_ 270, as described earlier. One of these models only includes O_3_ 270 as a solitary environmental variable. Finding PM_2.5_ and O_3_ in the majority of the top set lend strong support to their role in determining eczema severity. Finally, when looking at the double penalty approach for model selection, this resulted in one model that includes only O_3_ 270, lending more support for the role of O_3_ as a factor in determining eczema severity.

### Reproducibility: Korean cohort

A previous paper from South Korea examined the short-term effects of air pollution and meteorological variable on eczema symptoms. Using the AIC, MAv model selection was performed for available pollutants (Figure 2b): PM_10_, NO_2_, and O_3_. PM_10_ 60, O_3_ 270, and NO_2_ 365 performed best in the MAv model. According to the AIC, for single pollutants, PM_10_ performed the best, followed by NO_2,_ then O_3_ (Supplementary Table S5).

**Figure 5:**
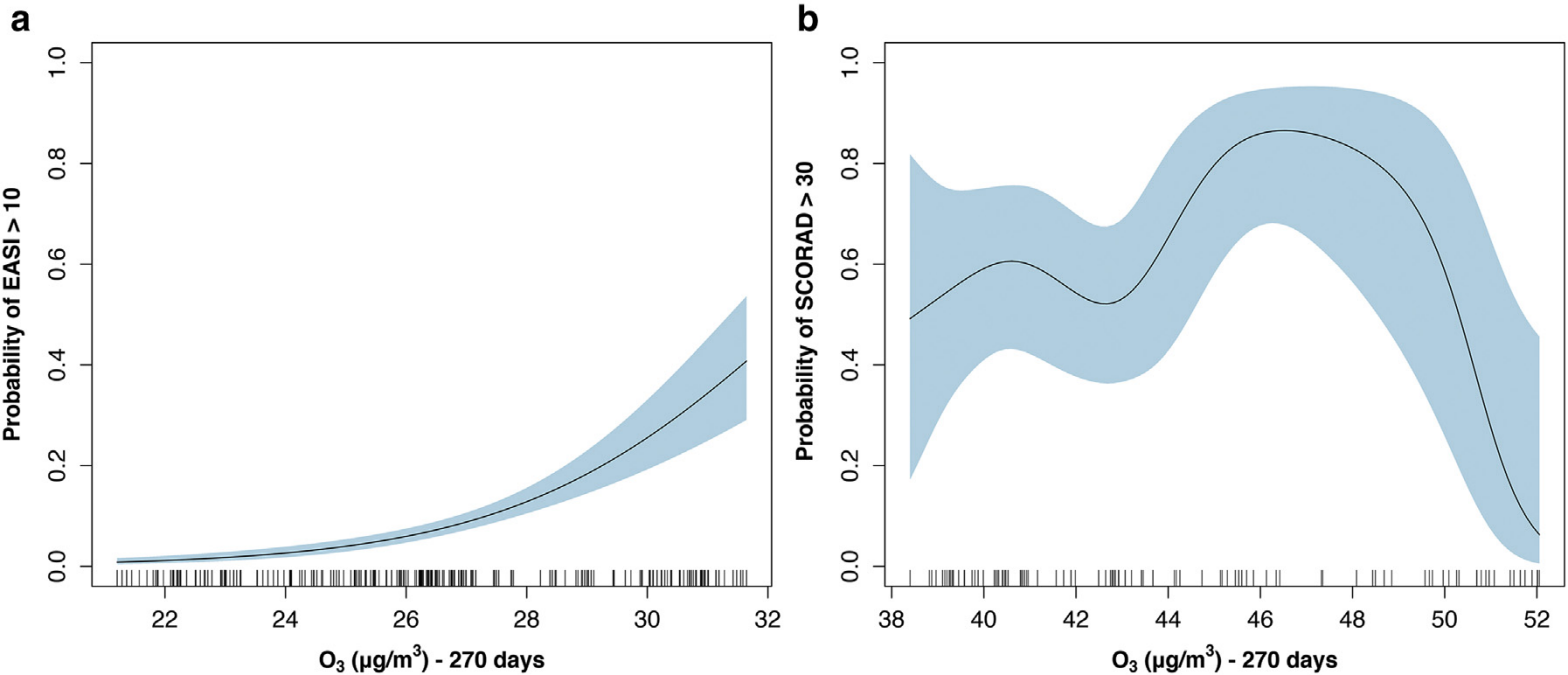
**Ground level O_3_ average over 270 days – THEA cohort and****Kim et al. (2017)****Korean Eczema Panel study.** O_3_ partial effects plot from (**a**) THEA cohort, n = 579 from 11 May 2018 to 10 March 2020 and the (**b**) Kim et al. (2017) Korean Panel study, n = 177 from 20 August 2013 to 14 June 2014. As age, sex and season were the only main features in common between cohorts, adjustments were only made to these features in both groups. Having these plots side by side demonstrate that they are extensions of each other with (**a**) showing lower average ozone levels and (**b**) showing the higher end of the spectrum. Wider 95% confidence interval for (**b**) as fewer cases studied. Probability of having an (**a**) EASI > 10 (y-axis) or a (**b**) SCORAD > 30 versus O_3_ 270 , O_3_ over 270 days (x-axis). The light blue shaded area represent the 95% confidence interval. The small dashes overlying the x-axis are known as the rug and they represent all of the cases. Note: Inability to draw a horizontal line throughout the 95% confidence interval indicates the smooth is significant. EASI, Eczema Area and Severity Index; O_3_, ozone; SCORAD, SCORing Atopic Dermatitis severity score; THEA, Tower Hamlets Eczema Assessment.

Using the same rules as with the East London dataset, we performed full model selection (Table 4). These models included only the NO_2_/PM_10_ composite and again O_3_ 270. Both variables in this model are significant and can be seen in Supplementary Figures S7 and S8. As values of the PM_10_/NO_2_ composite rise and O_3_ levels increase, the probability of having a SCORing Atopic Dermatitis (SCORAD) > 30 increases. For O_3_, at levels >46 μg/m^3^, the probability falls. When the O_3_ partial effect plots from our Tower Hamlets Eczema Assessment (THEA) cohort and the Korean groups were compared, they were almost extensions of each other (Figure 5).

**Table 4:** Top Model Set: Korea

Model Number	Equation	*R*^2^_Adj_	AIC		C-Index^1^ (95% CI)
**Base Model Before Backward Selection and Double Penalty Selection**					
Base	SCORAD30 ∼ NO_2_ 365/PM_10_ 60 Dim 1 + O_3_ 270 + Age + Sex + Season	0.122	228.53		0.76 (0.69–0.83)
**Two Best Supported Models Backward Selection (Δ6 AIC and Nesting Rule)**				**ΔAIC**	
3c	SCORAD30 ∼ NO_2_ 365/PM_10_ 60 Dim 1 + O_3_ 270	0.368	221.66	0	0.74 (0.67–0.83)
1d	SCORAD30 ∼ NO_2_ 365/PM_10_ 60 Dim 1 + O_3_ 270 + Age + Season	0.365	227.13	5.47	0.76 (0.69–0.83)

Abbreviations: 95% CI, 95% confidence interval; AIC, Akaike Information Criterion; NO_2_ 365, average level of nitrogen dioxide over the last 365 days; NO_2_ 365/PM_10_ 60 Dim 1, Dimension 1 of PCA between NO_2_ 365 and PM_10_ 60; O_3_ 270, average level of Ozone over the last 270 days; PM_10_ 60, average level of PM_10_ over the last 60 days.

1 C-Index; Concordance statistic, measure of goodness of fit, equal to the area under the receiver operating characteristic curve.

## Discussion

### Biologic rationale for findings

In this modeling study, we show that prolonged exposure to O_3_ rather than shorter time periods associate best with the severity of atopic eczema in the Bangladeshi population in East London. Full model selection left us with a top set of nine models, eight of which included O_3_ as the only environmental variable (plus a ninth, model 9, a possible O_3_ surrogate). An explanation for the selection of long-term O_3_ (O_3_ 270) exposure levels may be due to the protective nature of the lipid-rich layers of corneocytes (stratum corneum), which would include squalene, the single most abundant O_3_-reactive skin lipid (Wisthaler and Weschler, 2010). Long-term O_3_ exposure at increased levels may be required to overwhelm the protective mechanisms (Chen et al., 2007; Valacchi et al., 2005). This is in contrast to the lungs, which are known to react to short exposures to O_3_ and have simple columnar/cuboidal and thin squamous layers in the bronchioles and alveoli, respectively.

PM_2.5_ appears in eight models, although the raw data do not show a convincing direct relationship (Supplementary Figure S9). The eczema phenotype that develops in mice after exposure to diesel exhaust particles (Hidaka et al., 2017) supports its inclusion in the top set. We do see from our models that PM_2.5_ likely acts as an important O_3_ regulator. When at low levels, as seen in the PM_2.5_ partial effects plot, there is an increased probability of EASI >10. This is probably due to a reduction in consumption of O_3_ precursors by available PM_2.5_, with a resultant increase in O_3_ (Figure 2a). There is an increased probability of EASI > 10 with higher levels of PM_2.5_, particularly noticeable in model 6. It has been shown in mice that lung exposure to ultrafine PM (<0.1 μm) leads to little change, but the addition of O_3_ results in greater damage, possibly through the degradation of the PM to more volatile compounds (Wong et al., 2018).

### Interactions between ambient environmental variables

Model 9 is unusual because it does not contain O_3_ but retains NO/NO_2_, PM_10_/wind, temperature/humidity, and PM_2.5_. The presence of this combination of variables in one well-supported candidate model indicates a potential role for other atmospheric pollutants as well as O_3_. This model support is considerably weaker than for O_3_, with only the temperature/humidity composite showing a significant relationship. A review of the raw data and their relationship with change in EASI score suggests that these variables have less influence (Supplementary Figures S4 and S9 and Figure 2). O_3_ levels clearly start to rise in line with the increase in EASI scores seen at the end of the recruitment timeline, unlike other variables. Interestingly, when we use NO, wind speed, humidity, and PM_2.5_ using the MAv selected by the initial EASI 10 models, we are able to predict trends in O_3_ 270. It may be that the association that these variables have with eczema severity may not be direct but by their ability to alter O_3_ levels, for example, PM_2.5_ reactions with O_3_ precursors leading to lower production or NO reacting with O_3_ leading to increased consumption and subsequent lower O_3_ levels (Li et al., 2019a; Ma et al., 2016).

### Interpreting findings for Korean cohort

We also examined a dataset from Seoul, Korea that had eczema severity data for over 14 months using data from 76 monitoring stations in Seoul (Kim et al., 2017). The MAv model that best fits O_3_ and NO_2_ data was 270 and 365, respectively, the same MAv to those selected in the THEA cohort. Alongside being present in the top set from the Korean cohort, the fact that the O_3_ MAv (270 days) was the same across both datasets (Korea and London) and that the O_3_ partial effects plots aligned was very interesting, suggesting a relationship between eczema severity and O_3_ over a wide range of values from 22 to 32 μg/m^3^ in London and from 38 to 52 μg/m^3^ in Seoul. The recapitulation of similar findings is reassuring for our single station London model.

A review of pollution levels over the Korean study period shows that there was only one peak and trough, unlike the multiple cycles noted in the THEA cohort (Figure 2). The shorter time period in the Korean cohort is not long enough to reveal the underlying longer-term patterns. It is possible that if the recruitment process was continued through multiple cycles, O_3_ may be found to be more influential in the single-pollutant models. We found it surprising that at O_3_ 270 levels >46 μg/m^3^, the probability of having moderate or severe disease falls. An explanation could be cellular reduction‒oxidation adaptions from repeated exposure to high levels of O_3_ as shown in keratinocytes that were repeatedly exposed to cold plasma in vitro (the main constituent being O_3_) (Schmidt et al., 2016). An adapted response with some tolerance has also been noted in lungs exposed to very high (200 ppb/399.14 μg/m^3^) doses of O_3_ (Jörres et al., 2000).

### Clinical relevance

Our findings suggest that exposure to low levels of tropospheric O_3_ may be the most influential pollutant associated with the severity of eczema. In vitro evidence exists that O_3_-related oxidative and cytotoxic effects in keratinocytes can be reduced/repaired by the application of vitamin C compounds (Valacchi et al., 2015) and a proprietary snail mucus called HelixComplex (Gentili et al., 2020). This suggests that topical therapy as a preventative measure or treatment for O_3_ related flaring could be a new therapeutic approach.

It has been previously shown that pollutants, including O_3_, are associated with more visits to the emergency department (Wang et al., 2021) and an increase in eczema symptoms (Kim et al., 2017). If O_3_ levels do indeed have the strongest association with eczema severity, it may be that O_3_ is also driving flares and exacerbation in symptoms. Finding treatment options, such as those described earlier, to protect from the effects of O_3_ should be explored.

Finally, further work is required because epidemiological data suggest that there may be an association between eczema prevalence and O_3_ levels. We estimate the prevalence of eczema in Bangladeshi children in East London to be similar to that of the general population at around 16% (Ban et al., 2018; Williams et al., 1995). The rate seen in Bangladesh is 6‒12% of children (Ahmed et al., 2010; Kabir et al., 2005). We reviewed published pollution data in Dhaka, Bangladesh from 2013 to 2017. We found that average yearly tropospheric O_3_ levels were much lower in Dhaka than that seen in East London: 18.4 (standard error [SE]: ±0.68) μg/m^3^ versus 26.2 (SE: ±0.06) μg/m^3^. In addition, the PM_10_ and PM_2.5_ were markedly higher in Dhaka than in London: 154.9 (SE: ±2.59) and 85.8 (SE: ±1.59) μg/m^3^ versus 22.8 (SE: ±0.09) and 13.5 (SE: ±0.02) μg/m^3^, respectively.

Furthermore, in Korea, the prevalence of itchy ever eczema in individuals aged 6‒7 years has increased from 17 to 27% from the year 2000 to 2010 (Park et al., 2016). From 1999 to 2016 in Seoul, Korea, there was a 17.5 μg/m^3^ decrease in PM_10_ and a 17.6 μg/m^3^ increase in O_3_ (Kim and Lee, 2018; Seo et al., 2018). Over this period, solar irradiation, temperature, and humidity remained stable, and only wind speed increased (Seo et al., 2018). This is thought to be one explanation to account for some of the changes in PM_10_ and O_3_ (Seo et al., 2018). It is also important to note that PM_2.5_ fell significantly over this period (Kim and Lee, 2018). This reveals interesting information about the relationships between eczema prevalence and the ambient environment. If the relationship between O_3_ and eczema is real, we would expect to see more (severe) disease in the future as current (PM_2.5_ and NO_x_) pollution reduction strategies are employed and worldwide temperatures increase.

### Strengths and limitations

All data were carefully collected with detailed phenotyping, allowing us to accurately classify the disease and severity. The longer duration of the study period allowed the demonstration of longer trends in pollutant variables than single annual cycles. A final strength of the study was using the AIC with a ΔAIC of 6 to select a top model set rather than finding a minimal adequate model using null-hypothesis significance testing, which can lead to inflated type I errors (Harrison et al., 2018). One of the limitations of this study is that participants were only assessed during a single visit. As in any cohort study, some of the initial participants recruited may have had more severe eczema and may have been more likely to join at early stages, leading to a selection bias. This may account for the downward trend in EASI score over the study period–this would not however account for the uptick starting at the end of 2019. When we remove severe and clear patients from the dataset, the shape of severity remained the same, and outcomes from modeling remained unchanged.

This study uses one roadside monitoring station for its pollution data, and we do not have measured hyperlocal levels. Because O_3_ has become a more prominent pollutant, air quality monitoring networks with O_3_ measurements have been established in several countries in Europe, North America, Australia, Japan, and South Korea, and efforts have been made to regulate O_3_. There are 16 sites in London that record roadside pollution, with only one in East London. This is compared with 76 available stations in Seoul, Korea. We also do not account for indoor pollution, although there is some evidence that outdoor O_3_ levels strongly influence indoor levels, with the potential of indoor O_3_ levels exceeding those of outdoors (Huang et al., 2019).

In summary, these findings lend support to an important role of O_3_ in determining eczema severity in our cohort. The role of PM_2.5_ is important, although the strength of association is smaller and possibly secondary to ambient O_3_ levels. Further research on the relationship of ground-level O_3_, eczema, and its interplay with PM_2.5_ should be a focus. Mapping biological pathways and working out how to protect the skin from the effects of air pollutants would be a sensible next step to give clarity on how much influence it has on this very common disease. Future work on an analysis of global trends in pollution parameters and trends in the prevalence of eczema should also be undertaken.

## Materials and Methods

### Regulatory approvals

Recruitment for this cross-sectional study occurred between 11 May 2018 and 10 March 2020. Ethical approval was obtained from Health Research Authority, United Kingdom after review by Hampstead Regional Ethics Committee (reference: 18/LO/0018; Research Database Application (ReDA) Reference: 011978). Before recruitment and donation of samples, patients and/or parents gave their written informed consent. We followed the Strengthening the Reporting of Observational Studies in Epidemiology reporting guideline.

### Study and subjects

The participants were recruited as part of the THEA project, a study of atopic eczema in the Bangladeshi population in East London. All have a diagnosis of eczema, confirmed by Consultant Dermatologists, and are seen at the Royal London Hospital (London, United kingdom) for their condition. They all live in East London (Supplementary Figure S1) and are aged <31 years. Patients with known congenital recessive and X-linked ichthyoses, equivocal diagnoses of eczema, or mixed ethnicities were excluded from the study.

### Outcome

As a measure of severity, we used the EASI score (Schmitt et al., 2014). The primary outcome of this study was the probability of having an EASI score > 10 associated with previous exposure to pollutants and meteorological variables. To aid in the end-user interpretation of our models, we have dichotomized the EASI score into EASI 10 (EASI ≤ 10 and EASI > 10), which would translate into clear/mild versus moderate/severe.

We have chosen the EASI 10 cut-off using data previously generated from the cohort. For this, clustering was performed on binary eczema distribution data (e.g., eczema on left extensor elbow? Yes/No) in the first 409 participants in this cohort. K-means clustering using Manhattan distance was used. The number of clusters (k value) was suggested by using the elbow method heuristic using within-cluster sum of squares, and we settled on k = 4. Waikato Environment for Knowledge Analysis machine learning toolkit (version 3.9; University of Waikato, Hamilton, New Zealand) was used for clustering. Binary distribution data for each cluster group were converted into proportional means so that it gave the proportion of participants that had eczema at a particular site within each cluster. These data were then mapped onto body silhouettes to create a body heatmap for each cluster. We see that participants in cluster 1 are clear or almost clear, cluster 2 has the classic flexural disease, cluster 3 has extensor disease, and cluster 4 has extensive lesional coverage fitting with patterns that we see in the clinic (Supplementary Figure S2a).

When plotting EASI score (y-axis) by any variable (Supplementary Figure S2b), we use O_3_ averages over 365 days and color participants by cluster membership, and we can fit parsimonious demarcation lines between cluster 1 (clear or almost clear) and clusters 2‒4 at an EASI score of 10.

Previous studies have created severity strata (clear, mild, moderate, and severe) for the EASI score (Chopra et al., 2017a; Leshem et al., 2015). The kappa coefficient for gestalt subjective Investigators Global Assessment (IGA) and EASI in the Chopra et al. (2017a) study was 0.69, meaning that 31% of data were not concordant. The EASI range for moderate eczema was classified as 6.0‒22.9. When the kappa is manually calculated, using supplementary raw data (Chopra et al., 2017a) for agreement between moderate IGA and EASI between 6.0 and 22.9 versus other EASI/IGA scores, the kappa is 0.57. As the authors state, their severity strata are possible potential thresholds and by no means final. An EASI of 10 falls at the lower end of the moderate strata from the proposed strata in the Chopra et al. (2017a) paper. In a similar paper by Leshem et al. (2015), EASI and IGA have a kappa of 0.75, with the range of EASI of 7.1‒21.1 for moderate disease. In the Lesham paper, there is significant overlap between EASI and mild and moderate IGA, with an approximate minimum, maximum, and median (25^th^ and 75^th^ percentiles) of 2, 10, and 5 (3‒6), respectively, for EASI in the mild IGA group. For moderate IGA, the minimum, maximum, and median (25^th^ and 75^th^ percentiles) EASI scores are approximately 3, 24, 10 (7‒14), respectively. This is probably where their 18% misclassification was seen (raw data not available). With this in mind, the EASI severity strata, particularly for moderate disease, although accepted, is not yet clear.

We therefore classify that an EASI ≤ 10 is clear/mild and that an EASI > 10 is moderate or severe without the need for using the nonstandardized IGA as an anchor (Futamura et al., 2016).

We do not have a concern about dichotomizing the EASI score into clear/mild and moderate or severe because it is standard practice in dermatology to separate these groups apart for access to clinical trials and treatment. Intermediate inter-rater reliability in EASI scores (Schmitt et al., 2007), as seen in the seminal Hanifin et al. (2001) paper where one rater was excluded from the analysis to improve the results, still displays large differences in EASI score. This finding makes using EASI as a continuous variable unreliable. In addition, power issues seen with dichotomizing variables are largely a problem associated with dichotomizing explanatory variables (Altman and Royston, 2006).

### Statistical analysis/model selection

The probability of having an EASI > 10 depending on environmental variables was fitted using multivariable logistic GAMs while adjusting for potential confounders in the EASI > 10 and EASI ≤ 10 cohorts.

### Details of covariates used in models

Age and sex were recorded, and weight and height from the recruitment date were converted into body mass index. Participants’ postcodes were collected and converted into longitude and latitude. Longitude and latitude were used to adjust for any differences in the hyperlocal environment. The enrolment date was used to calculate the season at recruitment–the Solstices and Equinoxes being used as cut-offs. Social class was calculated using the European Socio-Economic Classification (ESeC) (Rose and Harrison, 2007). The ESeC score was collapsed into three groups: working class (ESeC 6‒9), middle class (ESeC 4‒6), and higher class (ESeC 1‒3) as per Rose and Harrison (2007).

Transepidermal water loss, a surrogate for skin barrier function, was measured using a Tewameter TM300 probe from Courage + Khazaka Electronics (Köln, Germany). Skin hydration of the epidermis was measured with the Corneometer CM825 probe, also from Courage + Khazaka Electronics. Both these measurements came from volar forearm nonlesional skin and an average of triplicate measurements was used. All measurements were performed on skin that was exposed to a climatically controlled environment for at least 20 minutes. Differences in the values of transepidermal water loss and skin hydration may represent underlying genetic variation resulting in defective skin barrier or could represent the effect of environmental factors directly impacting the barrier.

The investigator who performed the EASI assessment was recorded to allow adjustment for expected inter-rater variability in EASI scores.

### Weather/pollution stations and data quality control

Locally measured levels of wind speed (mph), relative humidity (%), and temperature (°C) and hourly concentrations of PM_10_ and PM_2.5_, NO, NO_2_, and tropospheric O_3_ were used in the analysis. Hourly measurements of wind speed (mph), relative humidity (%), and temperature (°C) were obtained from the Centre of Environmental Data Analysis web processing service (http://wps-web1.ceda.ac.uk/ui/home) using station source identification 18929 (London City Airport, United Kingdom), our nearest active station. All data used passed the Centre of Environmental Data Analysis’s quality control check. Values that did not pass were removed and imputed (see the section below). Precipitation was not recorded because the nearest active station with historic precipitation data was Reading (44.5 miles to the West of our study area). UV index data were also not collected for analysis because they were also not locally available. Hourly concentrations of PM_10_ and PM_2.5_, measured by Filter Dynamics Measurement System, and NO, NO_2,_ and tropospheric O_3_ were retrieved from Air Quality England (https://www.airqualityengland.co.uk/) using the Tower Hamlets–Blackwall (TH004) roadside monitoring dataset. This site was chosen because it was the nearest site that recorded O_3_ (Supplementary Figure S2). All pollutants were measured in μg/m^3^. All data used underwent quality assurance/control and was ratified (verified) by the Department of Environment, Food and Rural Affairs (London, United Kingdom). Data were collected from 1 October 2014 to 30 September 2020. We collected data from 4 years before the first participant recruitment to help to see the long-term trends and the relationships between the variables. All hourly data were converted into 24-hour average values.

### GAMs and top model set selection process

Because generalized linear models relax the assumption of normality required for ordinary least squares regression, GAMs take this further and can handle nonlinear relationships between the outcome and explanatory variables. GAMs are useful for modeling nonlinear data such as pollution and meteorological parameters and have been used extensively to investigate the association between health, (skin) disease, and the ambient environment (Kim et al., 2017; Ravindra et al., 2019; Wang et al., 2021).

In this paper, we use information theory rather than traditional null hypothesis significance testing. We have not used null hypothesis significance testing because a comparison of models using the likelihood-ratio test requires that the compared models are nested and all from the same parent model. Our approach uses the AIC, a metric that uses the maximum likelihood penalized by the number of model parameters, to assess model performance. The AIC itself is meaningless, but differences in AIC can be used to rank competing models, which can be non-nested, as many are in this paper. Multiple models are ranked, and a top set of possible explanatory models is selected using an AIC threshold to increase the probability of including the best-expected model (95% probability with an ΔAIC of 6) (Harrison et al., 2018). This approach assumes that multiple models (rather than only one in null hypothesis significance testing) may have the ability to explain a system equally well (Burnham et al., 2011). The presence of a variable in multiple models in the top set supports their inclusion in the system/model.

Logistic GAMs were used to analyze the nonlinear relationships between weather and pollution variables. Using R (version 3.5.3), we called the gam() function from the mgcv package (Wood, 2017; Wood, 2011). Because the outcome variable was binary, we used the binomial family argument. The smoothing parameter estimation method of restricted maximum likelihood and the AIC was used for model selection. Restricted maximum likelihood was chosen over generalized cross-validation owing to its superior performance and the ability to penalize overfitted models (Reiss and Todd Ogden, 2009; Wood, 2011). The mgcv package has been optimized for the AIC to function correctly with restricted maximum likelihood using the appropriate degrees of freedom/accounting for penalization (Wood et al., 2016).

Currently, there is no clear evidence of which exposure period and which level of a pollutant or meteorological variable has the strongest association with eczema severity. To understand this, we created nine MAvs for each meteorological and pollutant variable (average over the 7, 15, 30, 60, 90, 120, 180, 270, or 365 days preceding recruitment), which were also matched to the participants’ recruitment date to assess longer-term trends.

Per exploratory variable, the one model of 10 (value on the day of recruitment and nine MAv variables) with the lowest AIC was chosen to move forward to top model set selection. Simply put, the AIC is a number that can be used to compare models. It puts a value on how well models fit the data while penalizing complexity; lower values indicate improved support for the model (Burnham and Anderson, 2004, Burnham and Anderson, 2002).

The base model for meteorological and pollutant MAvs selection was as follows:

$$\text{Logit}\left(\text{E }\left[{\text{y}}_{\text{i}}\right]\right)={\text{f}}_{1}\left({\text{X}}_{1-10}\right)+{\text{f}}_{2}\left(\text{age}\right)+{\text{f}}_{3}\;\left(\text{body mass index}\right)+{\text{f}}_{4}\;\left(\text{longitude, latitude}\right)+{\text{f}}_{5}\left(\text{transepidermal water loss}\right)+{\text{f}}_{6}\left(\text{skin hydration}\right)+\text{factor }\left(\text{sex}\right)+\text{factor }\left(\text{season}\right)+\text{factor}\left(\text{investigator}\right)+\text{factor }\left(\text{ESeC}\right)\text{, }{\text{y}}_{\text{i}}∼\text{binary}$$

where E(y_i_) is EASI score >10 or ≤10, and X_1‒10_ are individual meteorological or pollutant variables and their nine associated MAvs. Per exploratory variable, the one model of 10 (value on the day of recruitment and nine MAv variables) with the lowest AIC was chosen to move forward to top model set selection.

It is well-established that climactic and pollutant data are often colinear (Supplementary Figure S3a), which can lead to problems with model fitting (Zuur et al., 2010). To avoid these problems, we generated composite variables in cases where variable pairs have correlation coefficients >0.8 (Dormann et al., 2013; Mateo et al., 2013). The variable pairs with the highest correlation undergo principal component analysis first, and dimension 1 is used as a composite. At this stage, the correlation analysis is repeated with the new composites and remaining variables until correlations between all variables are reduced below 0.8: in this case, NO 365 and NO_2_ 365, PM_10_ 270 and wind 365, and finally temperature 180 and humidity 180 are combined into three separate composites (Supplementary Figure S3b).

The base model for the top model set selection is as follows:

$$\text{Logit}\left(\text{E}\left[{\text{y}}_{\text{i}}\right]\right)={\text{f}}_{1}\left(\text{NO}/{\text{NO}}_{2}\text{ dimension }1\right)+{\text{f}}_{2}\left({\text{PM}}_{10}/\text{wind dimension }1\right)+{\text{f}}_{3}\left(\text{temperature }/\text{humidity}\;\right)+{\text{f}}_{4}\left({\text{PM}}_{2.5}\;90\right)+{\text{f}}_{5}\left({\text{O}}_{3}\;270\right)+{\text{f}}_{6}\left(\text{skin hydration}\right)+{\text{f}}_{7}\left(\text{transepidermal water loss}\right)+{\text{f}}_{8}\left(\text{age}\right)+{\text{f}}_{9}\left(\text{body mass index}\right)+{\text{f}}_{10}\left(\text{longitude, latitude}\right)+\text{factor }\left(\text{season}\right)+\text{factor }\left(\text{sex}\right)+\text{factor }\left(\text{investigator}\right)+\text{factor }\left(\text{ESeC}\right)\text{, }{\text{y}}_{\text{i}}\text{ ∼ binary}$$

In this model, f_1‒5_ represents the selected meteorological/pollutant variable with its associated MAv.

Two different approaches were used for model selection (Marra and Wood, 2011). The first approach was to use backward selection using the AIC. The top model set is usually a set based on the change in AIC from the top-performing AIC model (ΔAIC) (Burnham and Anderson, 2002). Traditionally, a ΔAIC of 2 was used for a top model set.

More recently, it has been shown that to ensure a 95% probability of having the best model included in the top model set, one should use a ΔAIC of 6 (Richards, 2008, 2005). When using the ΔAIC of 6, we used the nesting rule to help remove more complex but equally well-supported models within a ∼ Δ2 AIC (Arnold, 2010; Richards, 2008, 2005; Richards et al., 2011). Marra and Wood (2011) show that backward selection has low false-positive rates but can remove influential covariates, thereby increasing the false-negative rate, and so models were also selected using a second method, the double penalty approach. This approach adds an additional penalty to the smooth function and penalizes the functions that are only in the null space of the original penalty. The process essentially selects of the model variables that are not as influential. This approach can perform better than backward selection in terms of mean squared error, particularly when the data do not have high information content (Marra and Wood, 2011).

### Handling of missing data

In the weather dataset, 0.73% of the temperature and humidity data were missing, and 0.74% of the wind data were missing. In the Air Quality England pollution dataset, 25.80% of the PM_2.5_, 13.51% of the PM_10_, 6.44% of the O_3_, and 3.69% of the NO and NO_2_ data were absent. Multivariable imputation was performed for missing data. The multivariable approach using the mtsdi package was used (Junger and Ponce de Leon, 2015). The Expectation‒Maximization methods used have been shown to perform well on data sets with up to 40% missing values and even perform reasonably well with missing-not-at-random data. Performance for gaps in data of up to 7 days was also good. Of the methods available in the mtsdi package, the Expectation‒Maximization spline method was used because this has the greatest precision and accuracy. If missing values were present in other clinical variables, the case was removed from the analysis. A total of 25 cases were removed, leaving a total of 579 cases for analysis. Four cases lived completely out of the area. One case did not have recorded sex, one case did not have transepidermal water loss/skin hydration measurements, three cases did not have social class status, and 16 participants did not have height and weight recorded.

### Multiple testing

We have not corrected for multiple testing for two reasons. As an exploratory study, we did not want to increase our type II error and miss true positives in an attempt to improve type I error rates. The second reason is that the AIC was used for model selection and not *P*-values. We feel that this approach is free from the multiple testing problem (Burnham and Anderson, 2002).

### Sensitivity analysis

To ensure that our data were not reliant on underlying recruitment bias or sensitive to changes in data input, we checked for robustness by modifying the input dataset. We created three further truncated datasets (Supplementary Figure S4). We performed the same analysis for the main dataset and used the double penalty approach for top model selection–the resulting model outputs consistent with our main findings.

### Reproducibility

We used a dataset from a study published by the Allergy Department at the Samsung Medical Centre in Seoul, South Korea (Kim et al., 2017). This study looked at eczema symptoms in children aged 5 years or younger from August 2013 to December 2014 (<1 year) and the effects of meteorological conditions and pollutants on the presence of eczema symptoms. Data were available for 177 participants, including the patients’ date of enrolment, age, sex, season, and eczema severity as measured by another scoring criterion, SCORAD (Schmitt et al., 2014). SCORAD was only recorded on the day of enrolment; therefore, data from only this day were used.

We were able to access historic pollution data with O_3_, NO_2,_ and PM_10_ levels from Air Korea (https://www.airkorea.or.kr/). We collected data from the nearest monitoring station to the Samsung Medical Centre (Daechi-dong, Gangnam-gu, Seoul, Korea). The same data preparation and analysis described earlier for selecting single and full pollutant MAv models were used. Rather than EASI scoring, the SCORAD was used in this study. We dichotomized the SCORAD at 30 with one group >30 and one ≤30. SCORAD > 30 is comparable with an EASI > 10 when comparing the THEA and Korean EASI and SCORAD histograms and consulting the literature (Chopra et al., 2017a, 2017b). In a paper that reviews the severity stratification of eczema scoring methods, a SCORAD of 30, such as an EASI of 10, sits at the lower end of the moderate classification (Chopra et al., 2017a). PM_10_ 60 and NO_2_ 365 had a correlation coefficient of ‒0.85. PM_10_ 60 and NO_2_ 365 have correlation coefficients of ‒0.73 and ‒0.52 with O_3_ 270, respectively. Principal component analysis was performed to create a PM_10_/NO_2_ composite. The composite variable had a correlation coefficient of ‒0.65 with O_3_ 270.

The base model for the top model set selection in the Korean cohort was as follows:

$$\text{logit}\left(\text{E }\left[{\text{y}}_{\text{i}}\right]\right)={\text{f}}_{1}\left(\;{\text{PM}}_{10}/{\text{NO}}_{2}\;\right)+{\text{f}}_{2}\left({\text{O}}_{3}\;270\right)+{\text{f}}_{3}\left(\text{age}\right)+\text{factor }\left(\text{sex}\right)+\text{factor }\left(\text{season}\right)\text{, }{\text{y}}_{\text{i}}\text{∼ binary}$$

All data analyses were performed using R (version 3.5.3) and the mgcv for modeling and mtsdi for imputation.

### Data availability statement

Datasets related to this article can be found at https://figshare.com/ DOI: 10.6084/m9.figshare.12547400 (Thelwall and Kousha, 2016).

## ORCIDs

Bjorn R. Thomas: http://orcid.org/0000-0002-1815-3502

Xiang L. Tan: http://orcid.org/0000-0001-7685-7033

Shagayegh Javadzadeh: http://orcid.org/0000-0003-2415-607X

Elizabeth J. Robinson: http://orcid.org/0000-0002-2494-7421

Bryan S. McDonald: http://orcid.org/0000-0003-2590-0809

Malvina A. Krupiczojc: http://orcid.org/0000-0002-6909-2033

Syedia R. Rahman: http://orcid.org/0000-0002-8694-5173

Samiha Rahman: http://orcid.org/0000-0002-9265-5391

Rehana A. Ahmed: http://orcid.org/0000-0002-6843-8290

Rubina Begum: http://orcid.org/0000-0002-1086-8642

Habiba Khanam: http://orcid.org/0000-0002-1277-3559

David P. Kelsell: http://orcid.org/0000-0002-9910-7144

Jonathan Grigg: http://orcid.org/0000-0003-3109-6028

Robert J. Knell: http://orcid.org/0000-0002-3446-8715

Edel A. O’Toole: http://orcid.org/0000-0002-4084-4836

## Conflict of Interest

EAO has received payment from Sanofi for being on their Expert Grant Panel in 2019 (money paid to the university), has received consultancy fees from Palvella Therapeutics and Kamari Pharma (money paid to the university), and has received research funding from Kamari Pharma and Unilever (money paid to the university). Funding from Palvella and Kamari Pharma is for rare disease work. JG reports personal fees from Hodge Jones & Allen Solicitors to provide medical reports to a coroners case related to air pollution and personal fees from GSK, Vifor Pharmaceuticals, Novartis, BV Pharma, and AstraZeneca, outside the submitted work. The remaining authors state no conflict of interest.

## Supplementary Material

XJIDI_INNOV-2021-0062.R2-Supplement
