# Supplementary material for: Modeling of Temporal Exposure to the Ambient Environment and Eczema Severity
Source: JID Innov. 2021 Oct 9;2(1):100062. doi: 10.1016/j.xjidi.2021.100062 (PMC8713123; doi:10.1016/j.xjidi.2021.100062)
Supplement: XJIDI_INNOV-2021-0062.R2-Supplement [file mmc1.docx]

**Supplementary Online Content**

Modelling of temporal exposure to the ambient environment and eczema severity

BR Thomas*^1,2^, XL Tan^1^, S Javadzadeh^1^, EJ Robinson^2^, BS McDonald^1,2^, MA Krupiczojc^1,2^, SR Rahman^1,2^, S Rahman^1,2^, RA Ahmed^1,2^, R Begum^1,2^, H Khanam^1,2^, DP Kelsell^1^, J Grigg^1,2^, RJ Knell^3^ & EA O’Toole*^1,2^

**Figure S1.** Map/Chloropleth of THEA recruitment area – East London (579 participants).

**Figure S2.** Eczema distribution clusters (A) and EASI 10 selection (B) cut off process

**Figure S3.** Variable correlation plot of all continuous variables with scatter plots and Pearson’s correlation coefficients

**Figure S4.** Four datasets for testing robustness/sensitivity analysis of model selection

**Figure S5.** Partial effect plots from models 2-9 in top set (A-H)

**Figure S6.** ROC curve of top performing model sensitivity and specificity in the prediction of EASI 10 (AUC [95% CI]: 87.0 [83.1, 90.8]; p-value <0.001)

**Figure S7.** Partial effects for smooth variables from Korean top model 1

**Figure S8.** Partial effects for smooth variables from Korean top model 2

**Figure S9.** Level of PM_10_ over the study period with associated EASI10 – scatter plots

**Table S1.** Best supported models selected from 10 model variable set using Δ6 AIC

**Table S2.** Initial moving average model AIC values

**Table S3.** Selected Moving Average Pollution and Weather values for top model selection

**Table S4.** Model output from models 2-9 in top set (A-H)

**Table S5.** Initial moving average model AIC values in Korean dataset

This supplementary material has been provided by the authors to give readers additional information about their work.

**Figure S1.** Map/Chloropleth of THEA recruitment area – East London (579 participants).

Yellow to red shade indicates wards where participants have been recruited from. Grey shaded areas are wards within the recruitment area where no patients in the study reside. Black dot – Royal London Hospital. Blue dot – Blackwall roadside pollution monitoring station. Red dot – London City airport weather station


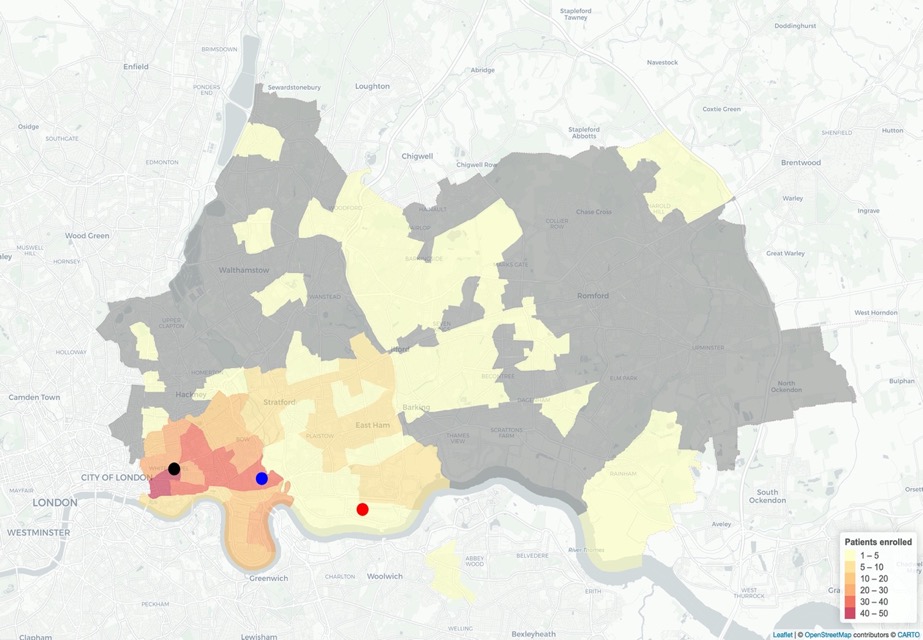


**1.0**

**0.0**


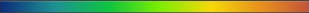

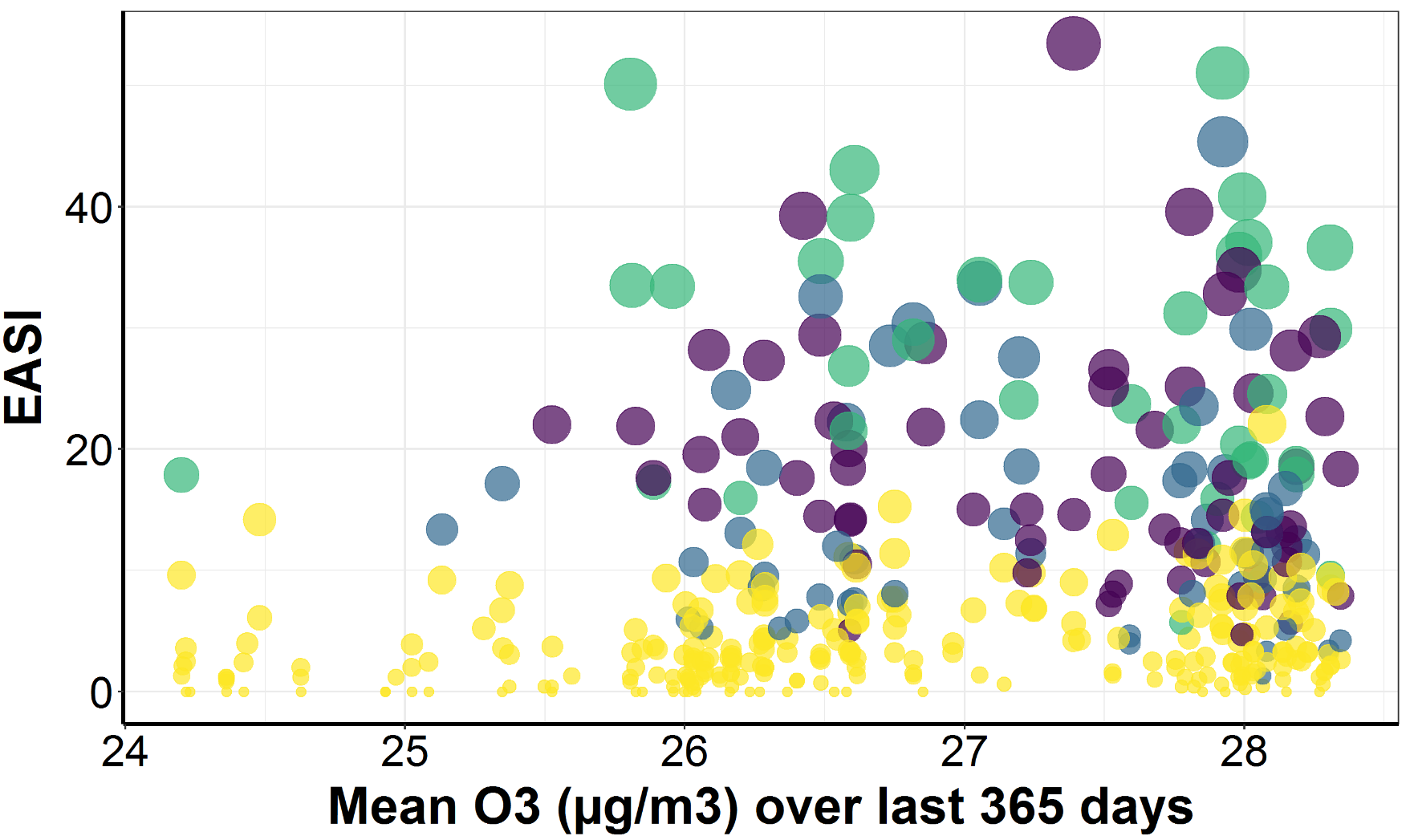


EASI 10

**A**

**Cluster 1 (n=254)**

**Cluster 2 (n=63)**

**Cluster 4 (n=37)**


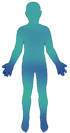

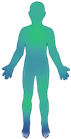

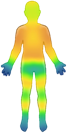

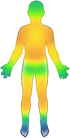

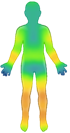

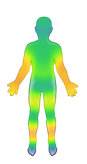

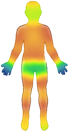

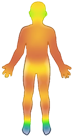


**Front**

**Back**

**Cluster 3 (n=55)**

**Cluster colour**

**B**

**Figure S2.** Eczema distribution clusters (A) and EASI 10 selection (B) cut off process

Eczema body site distribution heatmaps of four clusters (A). Front – front of body surface in anatomical position. Back – back of body in anatomical positions. Heatmap scale – 0.0 (blue): no participant has eczema at this site; 1.0 (red): all participants have eczema at this site. Cluster 1: Clear or almost clear; Cluster 2: Classic flexural disease; Cluster 3: Extensor pattern; Cluster 4: Extensive pattern. Cluster colour – cluster colour legend for EASI/O_3_ scatter plot (B). (B) represents an EASI scatterplot (n=409) using annual O_3_ as x-axis value. The colour of the points represents cluster membership as described in (A). The red horizontal line represent a parsimonious demarcation line between Cluster 1 (Clear or Almost Clear) and Clusters 2-4. The red line sits at an EASI score of 10.


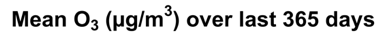


**Figure S3.** Variable correlation plot of all continuous variables with scatter plots and Pearson’s correlation coefficients

*Central diagonal from top-left to bottom-right* – Variable names. *Bottom left half of panel* – variable scatter plot with red line of best fit. *Top right half of panel* – Pearson’s correlation coefficient. *Values on edge of the entire plot* – Variable value range. (A) all continuous variables pre-analysis (B) final model meteorological and pollutant variables.

EASI10, EASI score > 10; Wind 365, average daily mean wind speed over the last 365 days; Temp 180, average daily mean temperature over the last 180 days; Hum 180, relative humidity over the last 180 days; PM_10_ 270, average level of PM_10_ over the last 270 days; PM_2.5_ 120, average level of PM_2.5_ over the last 120 days; NO 365, average level of nitric oxide over the last 365 days; NO_2_ 365, average level of nitrogen dioxide over the last 365 days; O_3_ 270, average level of ozone over the last 270 days; PM_2.5_*,* 2.5µm particulate matter; TEWL, transepidermal water loss as measured by a Tewameter; SH, Skin hydration as measured by a corneometer; NO/NO2, Dimension 1 of PCA between NO 365/NO_2_ 365; PM_10_/Wind, Dimension 1 of PCA between PM_10_ 270, Wind 365; Temp/Hum, Dimension1 of PCA between Temp 180 and Hum 180; PCA, Principal component analysis

**A**

**B**


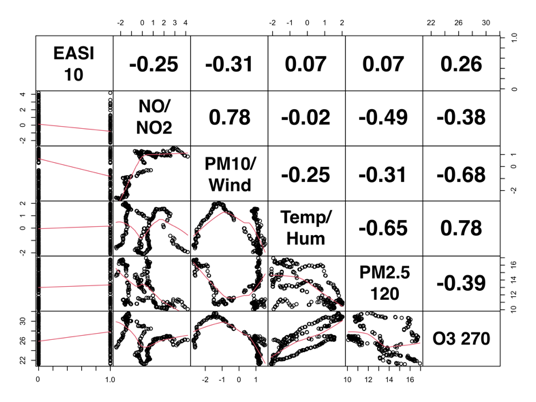

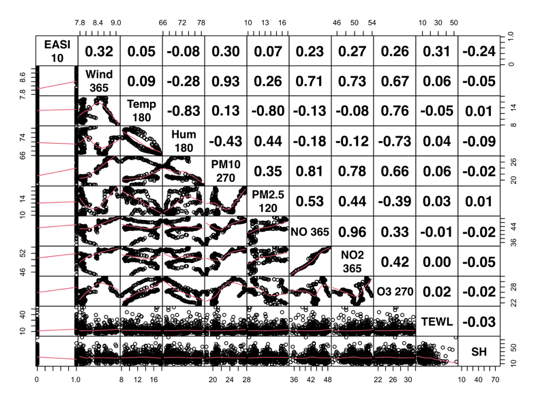


**Figure S4.** Four datasets for testing robustness of model selection

Four panels demonstrating the EASI score (0-72) over the entirety of the enrolment period. Panel A shows the full dataset used in the main analysis. Panel B represents a dataset where patients must have lesional eczema (i.e. cannot be clear) or if they are clear, they must be on immunosuppressive medication. Panel C is a dataset where participants cannot be clear and must be using topical steroids on the face and body or must be on immunosuppressants. Finally, panel D shows participants that have the features of dataset (C) and cannot have very severe eczema (EASI < 20; Max 72). The blue line represents the locally estimated scatterplot smoothing (LOESS) trends for EASI score over time. The 95% CIs from the LOESS estimate are shown as the shaded grey area.


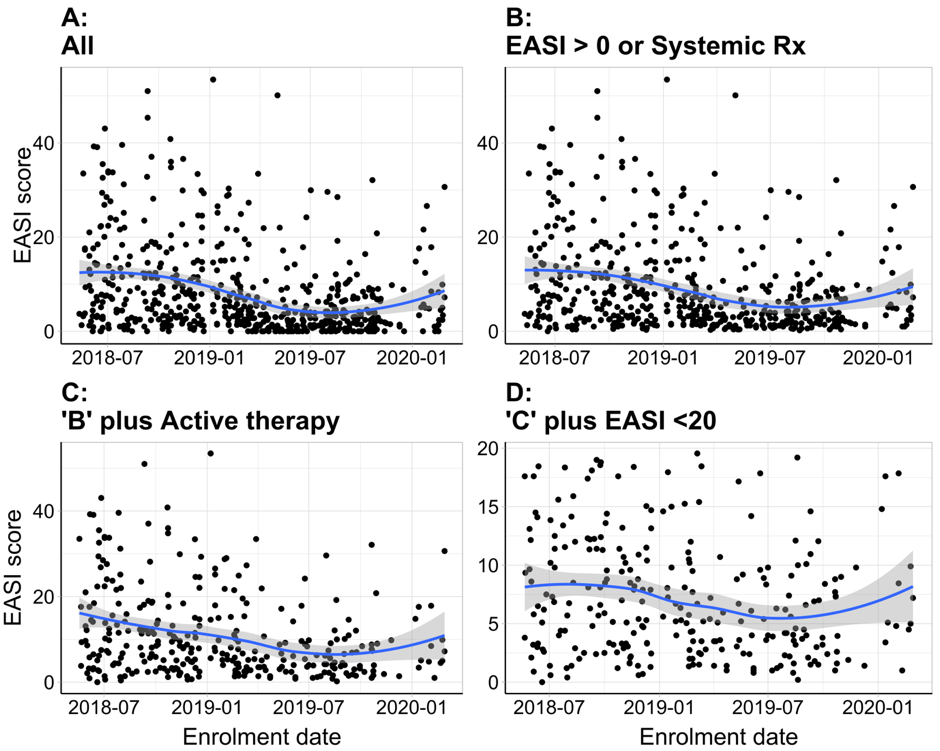


**Figure S5.** Partial effect plots from models 2-9 in top set (A-H)

**Figure S5 A. Top model (2): Smooth terms (non-linear parameters) partial effects plots: O_3_ and PM_2.5_ versus probability of EASI > 10**

Probability of having an EASI > 10 (y-axis) dependent on the x-axis values: (A) PM_2.5_ over 120 days (p = 0.148), (B) ozone over 270 days (p = <0.001), (C) skin hydration measured using a corneometer (p = <0.001), (D) TEWL measured using a tewameter (p = <0.001) and (E) age (p= <0.001).

The light blue shaded area represent the 95% confidence interval. The small dashes overlying the x-axis are known as the ‘rug’ and they represent all of the cases. *Note*: if you cannot draw a horizontal line throughout the 95% confidence interval indicating the smooth is indeed significant.

EASI, eczema area and severity index; AU, arbitrary units; TEWL, transepidermal water loss


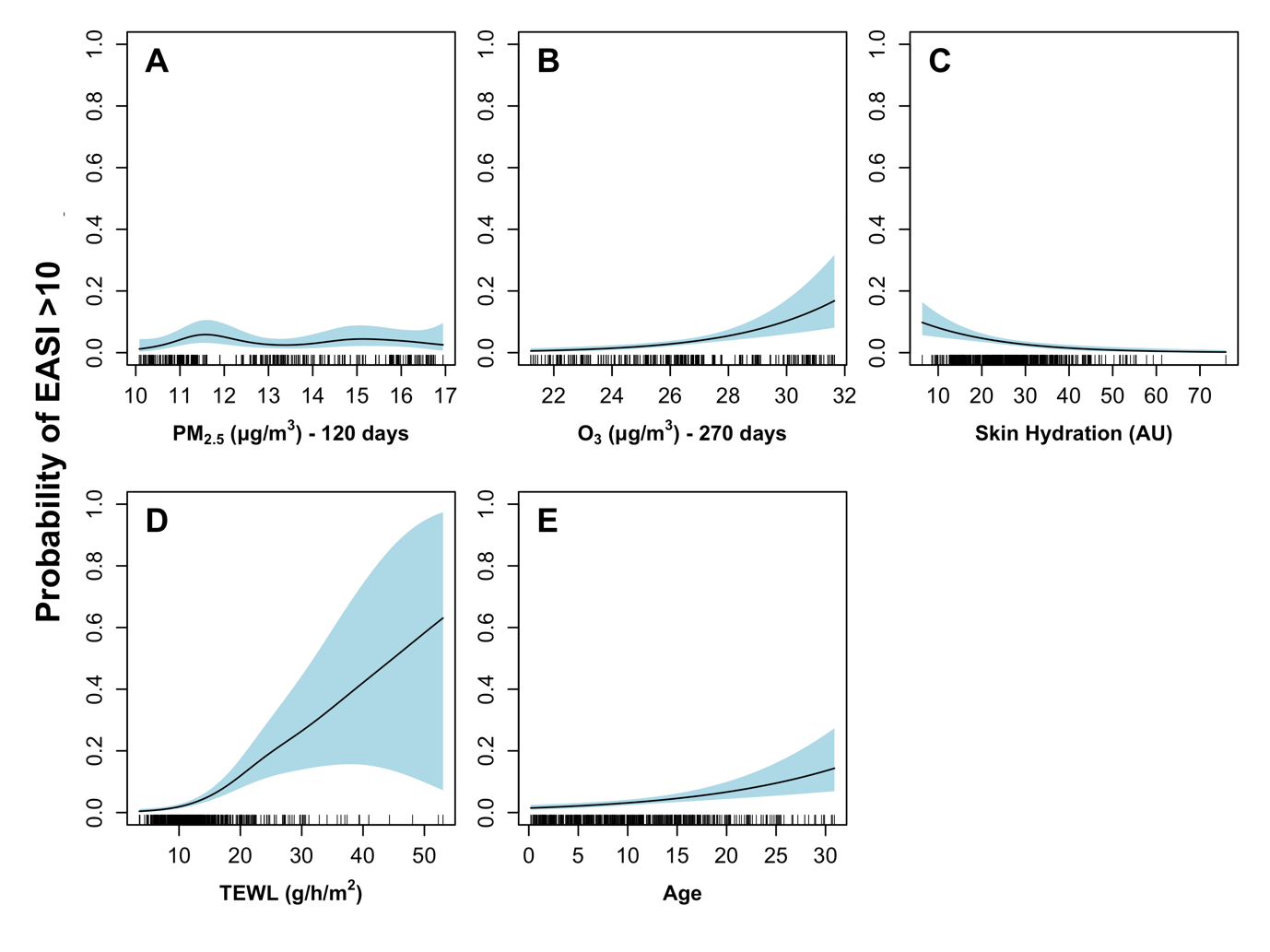


**Figure S5 B. Top model (3): Smooth terms (non-linear parameters) partial effects plots: O_3_ and PM_2.5_ versus probability of EASI > 10**

Probability of having an EASI > 10 (y-axis) dependent on the x-axis values: (A) PM_2.5_ over 120 days (p = 1.48), (B) ozone over 270 days (p = <0.001), (C) skin hydration measured using a corneometer (p = <0.001), (D) TEWL measured using a tewameter (p = <0.001), (E) age (p = <0.001) and (F) body mass index (p = 0.172).

The light blue shaded area represent the 95% confidence interval. The small dashes overlying the x-axis are known as the ‘rug’ and they represent all of the cases. *Note*: if you cannot draw a horizontal line throughout the 95% confidence interval indicating the smooth is indeed significant.

EASI, eczema area and severity index; AU, arbitrary units; TEWL, transepidermal water loss


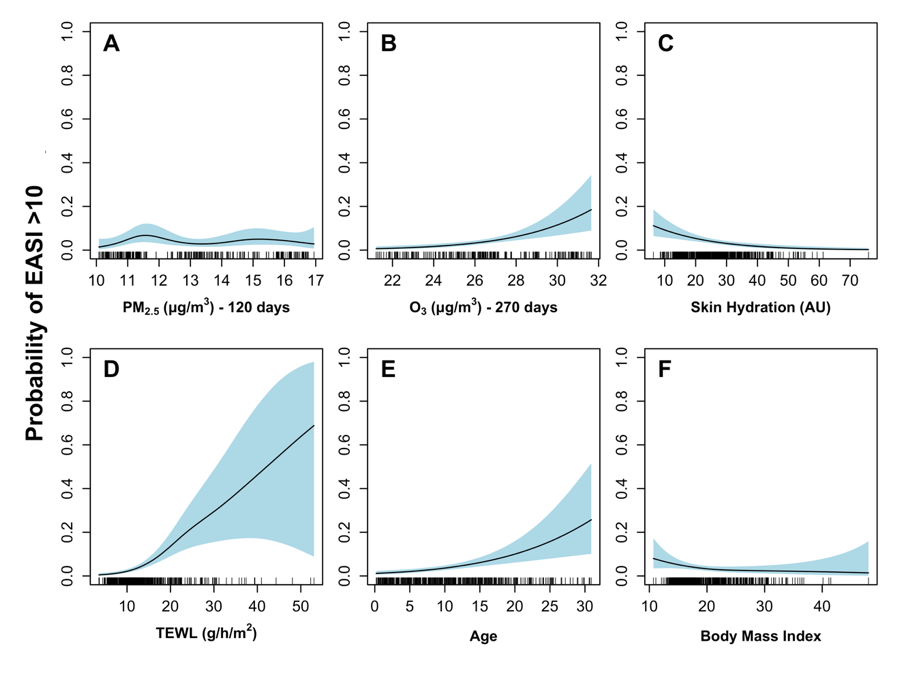


**Figure S5 C. Top model (4): Smooth terms (non-linear parameters) partial effects plots: O_3_ and PM_2.5_ versus probability of EASI > 10**

Probability of having an EASI > 10 (y-axis) dependent on the x-axis values: (A) ozone over 270 days (p = <0.001), (B) skin hydration measured using a corneometer (p = <0.001), (C) TEWL measured using a tewameter (p = <0.001), (D) age (p = <0.001) and (E) body mass index (p = 0.133).

The light blue shaded area represent the 95% confidence interval. The small dashes overlying the x-axis are known as the ‘rug’ and they represent all of the cases. *Note*: if you cannot draw a horizontal line throughout the 95% confidence interval indicating the smooth is indeed significant.

EASI, eczema area and severity index; AU, arbitrary units; TEWL, transepidermal water loss


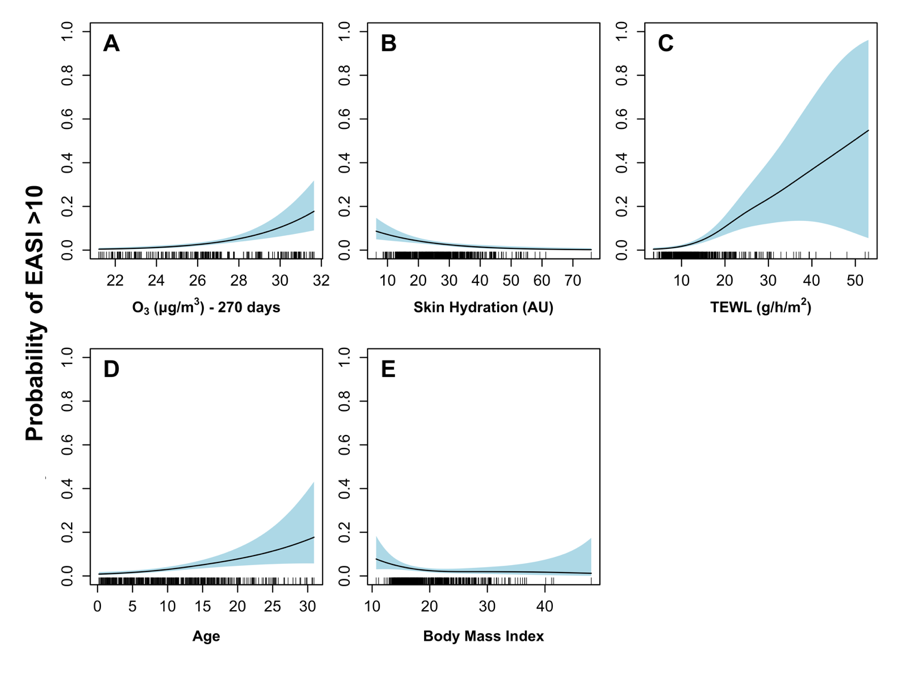


**Figure S5 D. Top model (5): Smooth terms (non-linear parameters) partial effects plots: O_3_ and PM_2.5_ versus probability of EASI > 10**

Probability of having an EASI > 10 (y-axis) dependent on the x-axis values: (A) PM_2.5_ over 120 days (p = 0.01), (B) ozone over 270 days (p = <0.001), (C) skin hydration measured using a corneometer (p = <0.001), (D) TEWL measured using a tewameter (p = <0.001), (E) age (p = <0.001) and (F) body mass index (p = 0.173).

The light blue shaded area represent the 95% confidence interval. The small dashes overlying the x-axis are known as the ‘rug’ and they represent all of the cases. *Note*: if you cannot draw a horizontal line throughout the 95% confidence interval indicating the smooth is indeed significant.

EASI, eczema area and severity index; AU, arbitrary units; TEWL, transepidermal water loss


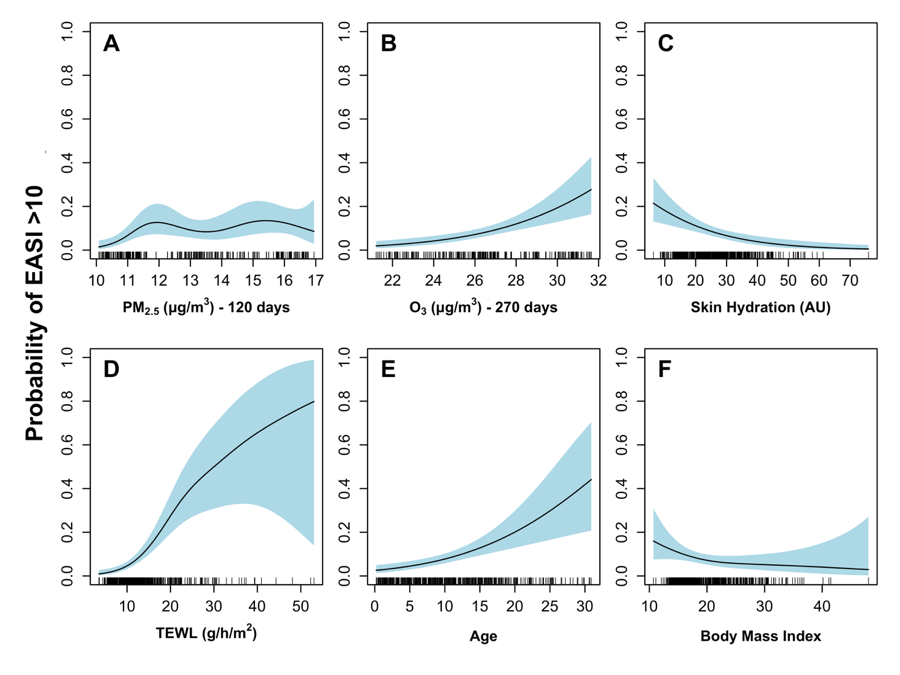


**Figure S5 E*.* Top model (6): Smooth terms (non-linear parameters) partial effects plots: O_3_ and PM_2.5_ versus probability of EASI > 10**

Probability of having an EASI > 10 (y-axis) dependent on the x-axis values: (A) Dimension 1 of PCA between NO 365/NO_2_ 365 (p = 0.176), (B) PM_2.5_ over 120 days (p = 0.083), (C) ozone over 270 days (p = 0.003), (D) skin hydration measured using a corneometer (p = <0.001), (E) TEWL measured using a tewameter (p = <0.001), (F) age (p = <0.001) and (H) body mass index (p = 0.148).

The light blue shaded area represent the 95% confidence interval. The small dashes overlying the x-axis are known as the ‘rug’ and they represent all of the cases. *Note*: if you cannot draw a horizontal line throughout the 95% confidence interval indicating the smooth is indeed significant.


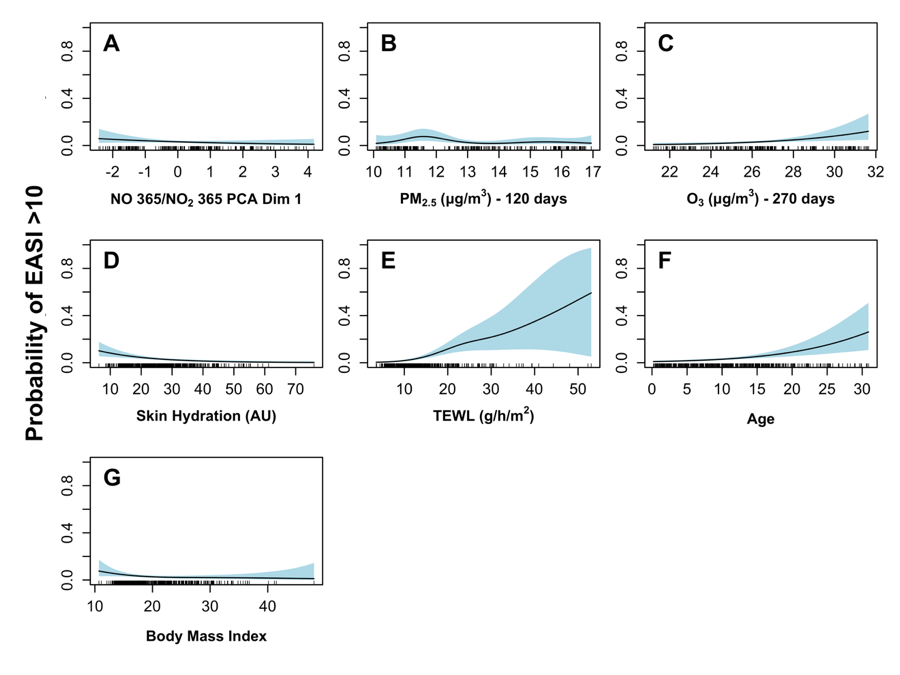


**Figure S5 F. Top model (7): Smooth terms (non-linear parameters) partial effects plots: O_3_ and PM_2.5_ versus probability of EASI > 10**

Probability of having an EASI > 10 (y-axis) dependent on the x-axis values: (A) PM_2.5_ over 120 days (p = 0.087), (B) ozone over 270 days (p = <0.001), (C) skin hydration measured using a corneometer (p = <0.001), (D) TEWL measured using a tewameter (p = <0.001), (E) age (p = <0.001) and (F) body mass index (p = 0.127).

The light blue shaded area represent the 95% confidence interval. The small dashes overlying the x-axis are known as the ‘rug’ and they represent all of the cases. *Note*: if you cannot draw a horizontal line throughout the 95% confidence interval indicating the smooth is indeed significant.

EASI, eczema area and severity index; AU, arbitrary units; TEWL, transepidermal water loss


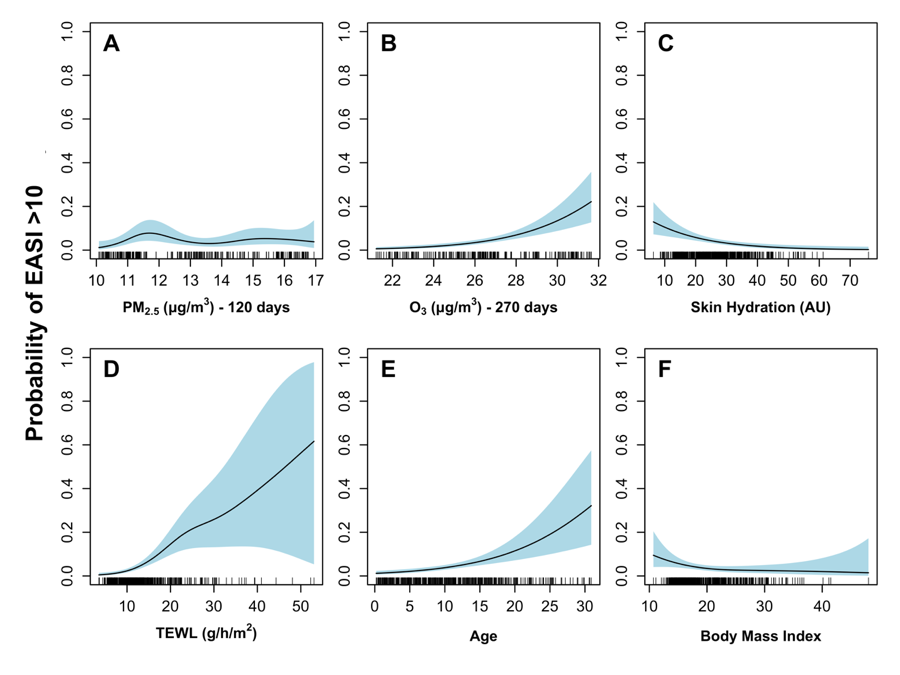


**Figure S5 G. Top model (8): Smooth terms (non-linear parameters) partial effects plots: O_3_ and PM_2.5_ versus probability of EASI > 10**

Probability of having an EASI > 10 (y-axis) dependent on the x-axis values: (A) PM_2.5_ over 120 days (p = 0.11), (B) ozone over 270 days (p = <0.001), (C) skin hydration measured using a corneometer (p = <0.001), (D) TEWL measured using a tewameter (p = <0.001), (E) age (p = <0.001) and (F) body mass index (p = 0.100).

The light blue shaded area represent the 95% confidence interval. The small dashes overlying the x-axis are known as the ‘rug’ and they represent all of the cases. *Note*: if you cannot draw a horizontal line throughout the 95% confidence interval indicating the smooth is indeed significant.

EASI, eczema area and severity index; AU, arbitrary units; TEWL, transepidermal water loss


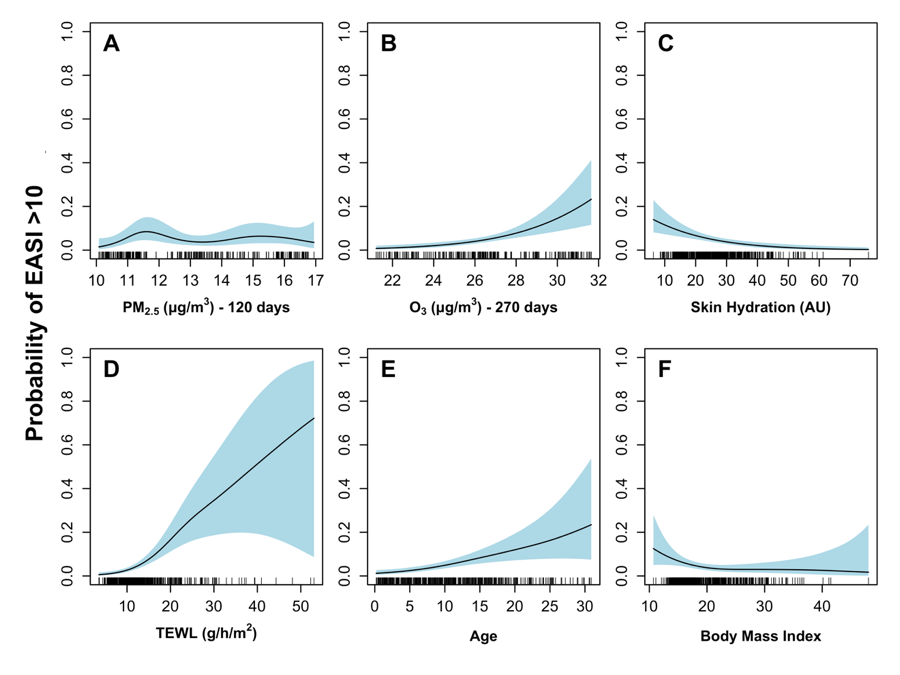


**Figure S5 H. Top model (9): Smooth terms (non-linear parameters) partial effects plots: O_3_ and PM_2.5_ versus probability of EASI > 10**

Probability of having an EASI > 10 (y-axis) dependent on the x-axis values: (A) Dimension 1 of PCA between NO 365/NO_2_ 365 (p = 0.669), (B) Dimension 1 of PCA between PM_10_ 270/Wind 365 (p = 0.122), (C) Dimension 1 of PCA between Hum 180/Temp 180 (p = 0.122), (D) PM_2.5_ over 120 days (p = 0.140), (E) skin hydration measured using a corneometer (p = <0.001), (F) TEWL measured using a tewameter (p = <0.001), (G) age (p = <0.001) and (H) body mass index (p = 0.188).

The light blue shaded area represent the 95% confidence interval. The small dashes overlying the x-axis are known as the ‘rug’ and they represent all of the cases. *Note*: if you cannot draw a horizontal line throughout the 95% confidence interval indicating the smooth is indeed significant.

EASI, eczema area and severity index; AU, arbitrary units; TEWL, transepidermal water loss


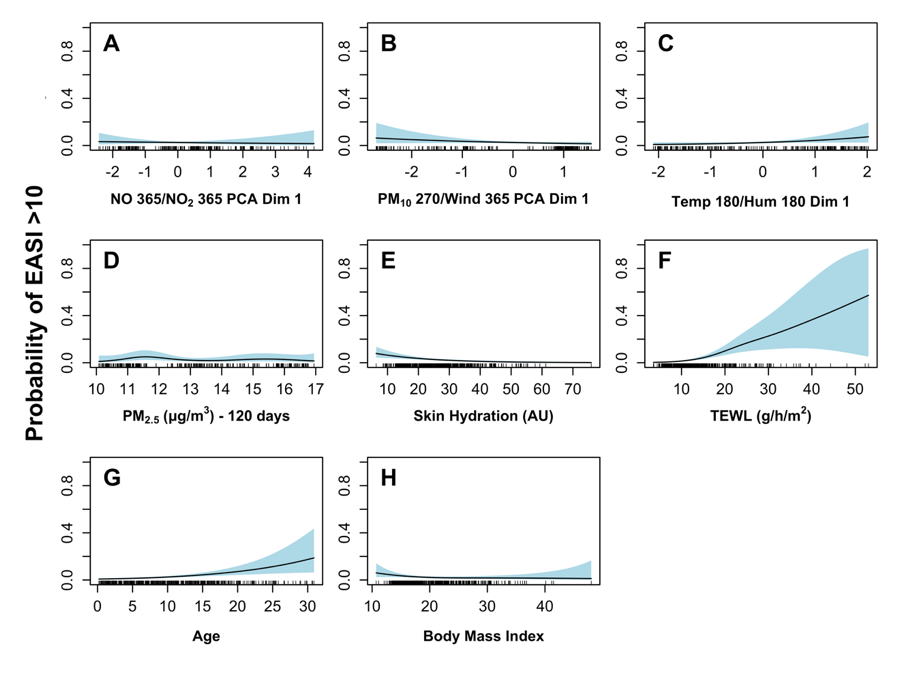

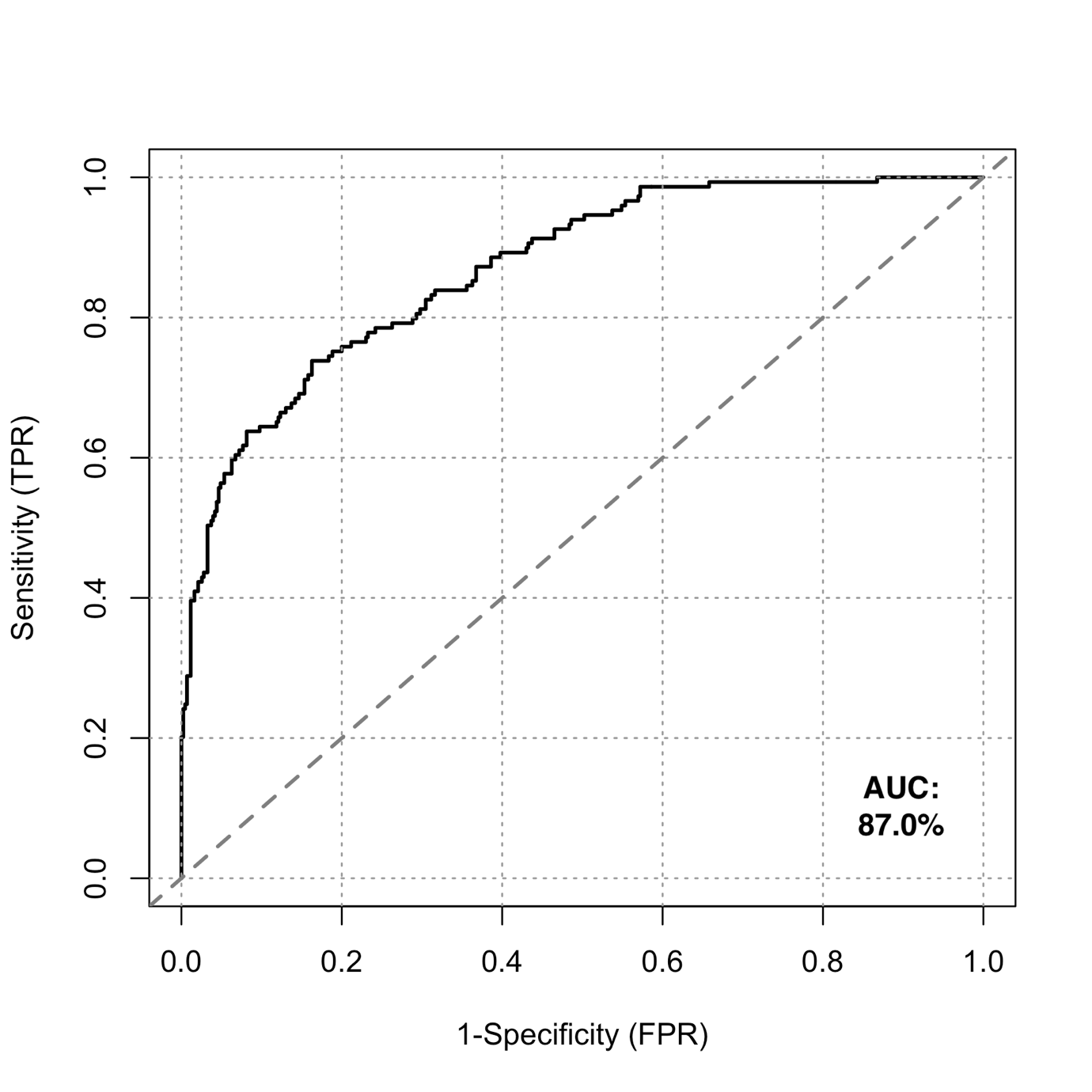


**Figure S6.** ROC curve of top performing model sensitivity and specificity in the prediction of EASI 10 (AUC/C-Index [95% CI]: 0.87 [0.83, 0.91]; p-value <0.001)

ROC Receiver operating curve; AUC, Area under the curve; TPR, True positive rate; FPR, False positive rate; C-Index, Concordance statistic

**AUC:**

**0.87**

**Figure S7.** Partial effects for smooth variables from Korean top model 1

Probability of having a SCORAD > 30 (y-axis) dependent on the x-axis values: (A) Dimension 1 of PCA between NO 365/PM_10_ 60, (B) Ozone over 270 days

The light blue shaded area represent the 95% confidence interval. The small dashes overlying the x-axis are known as the ‘rug’ and they represent all of the cases. *Note*: if you cannot draw a horizontal line throughout the 95% confidence interval indicating the smooth is indeed significant.

SCORAD, SCORing Atopic Dermatitis severity score; PCA, principal components analysis


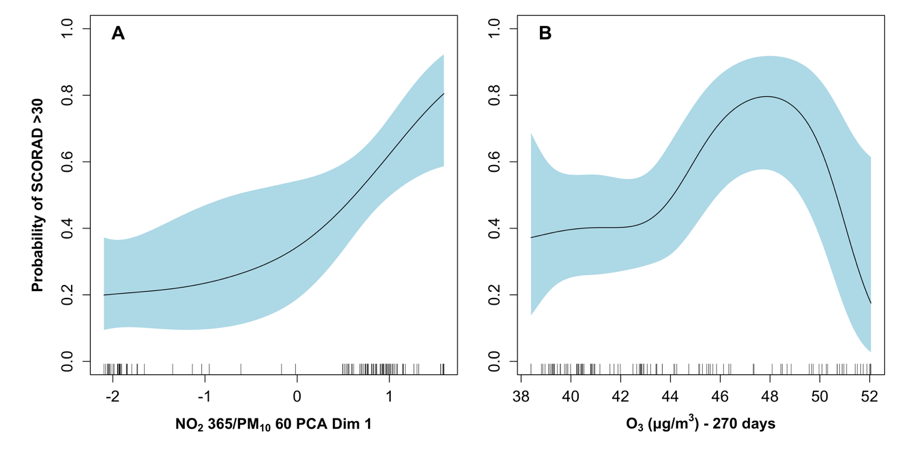


**Figure S8.** Partial effects for smooth variables from Korean top model 2

Probability of having a SCORAD > 30 (y-axis) dependent on the x-axis values: (A) Dimension 1 of PCA between NO 365/PM_10_ 60, (B) Ozone over 270 days and (C) age.

The light blue shaded area represent the 95% confidence interval. The small dashes overlying the x-axis are known as the ‘rug’ and they represent all of the cases. *Note*: if you cannot draw a horizontal line throughout the 95% confidence interval indicating the smooth is indeed significant.

SCORAD, SCORing Atopic Dermatitis severity score; PCA, principal components analysis


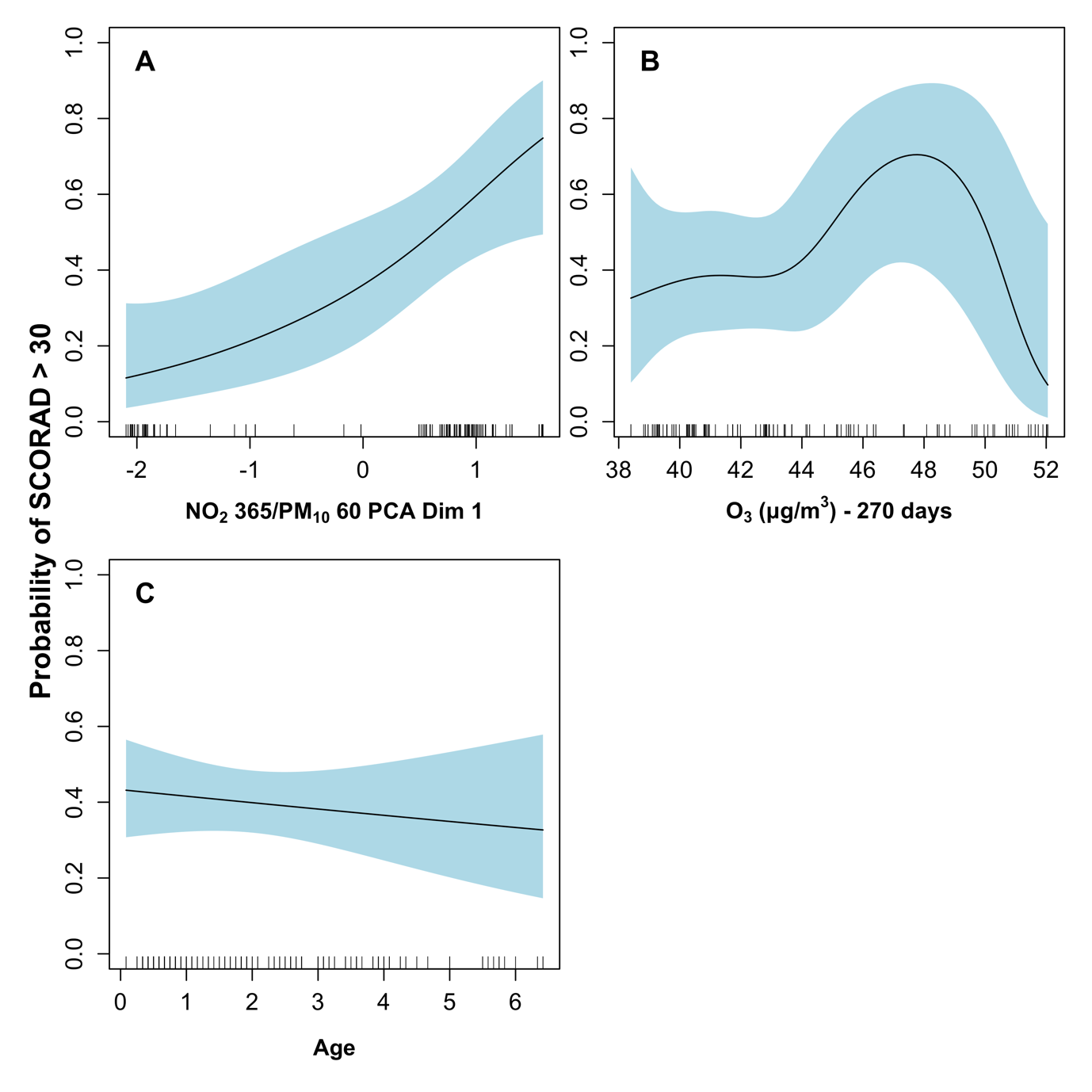

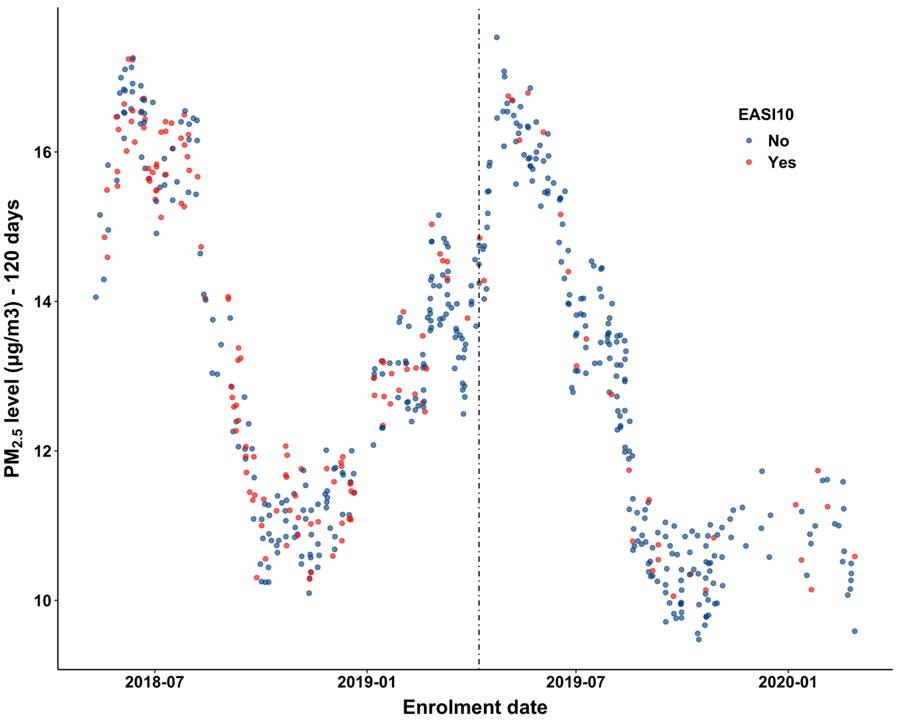


**Figure S9.** Level of PM_2.5_ over the study period with associated EASI10 – scatter plots

PM_2.5_ level averaged over 120 days (y-axis) and chronological enrolment date (x-axis) demonstrates the distribution of EASI score > 10 (red) and <= 10 (blue)

**Table S1.** Best supported models selected from 10 model variable set using Δ6 AIC

| **Selected Models** | **df:**  **Model** | **edf:**  **Specific variable** | ***R*^2^_Adj_** | **AIC** | **ΔAIC** |
| --- | --- | --- | --- | --- | --- |
| **O_3_ 270** | 22.62 | 1.00 | 0.351 | 481.95 | 0 |
| **PM_10_ 270**^a^ | 25.75 | 3.45 | 0.347 | 489.00 | 7.05 |
| **Wind 365** | 25.47 | 2.99 | 0.346 | 489.70 | 7.75 |
| **Hum 180**^a,b^ | 22.60 | 1.00 | 0.323 | 496.84 | 14.89 |
| **PM_2.5_ 120^a^** | 27.12 | 5.01 | 0.331 | 497.64 | 15.69 |
| **NO_2_ 365**^a,b^ | 22.57 | 1.00 | 0.319 | 500.22 | 18.27 |
| **Temp 180**^a^ | 23.54 | 1.22 | 0.321 | 500.40 | 18.45 |
| **NO 365**^a^ | 22.51 | 1.00 | 0.312 | 503.40 | 21.45 |
| Δ6 AIC, models with an AIC score of within 6 of the best-supported AIC model; df, degrees of freedom; edf, estimated degrees of freedom; *R*^2^_Adj_ , Adjusted *R*^2^*;* AIC, Akaike Information Criterion | | | | | |
| Models (name includes discriminatory variable of interest): Wind 365, average wind speed over the last 365 days; Temp 180, average daily mean temperature over the last 180 days; Hum 180, average level of relative humidity over the last 180 days; PM_10_*,* 10µm particulate matter; PM_10_ 270, average level of PM_10_ over the last 270 days; PM_2.5_*,* 2.5µm particulate matter; PM_2.5_ 120, average level of PM_2.5_ over the last 120 days; NO 365, average level of Nitric Oxide over the last 365 days; NO_2_ 365, average level of Nitrogen Dioxide over the last 365 days; O_3_ 270, average level of Ozone over the last 270 days | | | | | |
| a – Temp 60, 90, 120, 365, Hum 60, 270, PM_10_ 365, PM_2.5_ 270, NO on the day, 7, 60, 90, 120 & 180, NO_2_ 15, 30, 60, 90, all within Δ6 AIC of the ‘selected’ model (Supplementary 2). | | | | | |
| b – Hum 270 and NO_2_ 60 within Δ2 AIC of the ‘selected’ model | | | | | |

**Table S2.** Initial moving average model AIC values

| **Wind Models** | **Degrees of freedom** | **AIC** |
| --- | --- | --- |
| wind | 22.67 | 509.82 |
| wind7 | 22.64 | 509.57 |
| wind15 | 27.21 | 506.91 |
| wind30 | 22.55 | 506.78 |
| wind60 | 22.74 | 506.85 |
| wind90 | 24.95 | 508.73 |
| wind120 | 23.38 | 507.87 |
| wind180 | 22.63 | 509.83 |
| wind270 | 25.58 | 504.89 |
| wind365 | 25.47 | 489.70 |
| **Temperature Models** | **Degrees of freedom** | **AIC** |
| temp | 22.65 | 509.82 |
| temp7 | 22.63 | 509.83 |
| temp15 | 22.49 | 508.22 |
| temp30 | 22.53 | 508.81 |
| temp60 | 25.64 | 504.78 |
| temp90 | 25.60 | 503.41 |
| temp120 | 23.54 | 502.57 |
| temp180 | 23.54 | 500.40 |
| temp270 | 24.52 | 506.91 |
| temp365 | 23.92 | 506.32 |
| **Relative Humidity Models** | **Degrees of freedom** | **AIC** |
| hum | 22.86 | 508.73 |
| hum7 | 22.47 | 509.51 |
| hum15 | 23.46 | 509.74 |
| hum30 | 23.92 | 509.06 |
| hum60 | 26.08 | 502.82 |
| hum90 | 25.54 | 506.08 |
| hum120 | 22.59 | 506.43 |
| hum180 | 22.60 | 496.84 |
| hum270 | 24.79 | 497.10 |
| hum365 | 22.61 | 505.25 |
| **<10µm Particulate Matter Models** | **Degrees of freedom** | **AIC** |
| pm10 | 22.63 | 509.78 |
| pm107 | 22.42 | 509.06 |
| pm1015 | 22.56 | 509.56 |
| pm1030 | 22.58 | 509.73 |
| pm1060 | 22.53 | 509.23 |
| pm1090 | 23.58 | 509.64 |
| pm10120 | 26.10 | 504.31 |
| pm10180 | 22.34 | 502.32 |
| pm10270 | 25.75 | 489.00 |
| pm10365 | 22.52 | 493.27 |

**Table S2.** Initial moving average model AIC values (continued)

| **<2.5µm Particulate Matter Models** | **Degrees of freedom** | **AIC** |
| --- | --- | --- |
| pm25 | 22.61 | 509.83 |
| pm257 | 22.60 | 509.81 |
| pm2515 | 23.12 | 510.27 |
| pm2530 | 22.49 | 509.04 |
| pm2560 | 22.44 | 508.05 |
| pm2590 | 26.81 | 504.26 |
| pm25120 | 27.12 | 497.64 |
| pm25180 | 22.44 | 508.57 |
| pm25270 | 26.75 | 503.19 |
| pm25365 | 26.03 | 509.02 |
| **Nitric Oxide Models** | **Degrees of freedom** | **AIC** |
| no | 23.81 | 507.79 |
| no7 | 23.45 | 508.56 |
| no15 | 24.70 | 509.81 |
| no30 | 22.95 | 510.06 |
| no60 | 24.33 | 509.39 |
| no90 | 24.84 | 508.34 |
| no120 | 24.76 | 506.10 |
| no180 | 24.28 | 506.71 |
| no270 | 24.58 | 510.04 |
| no365 | 22.51 | 503.41 |
| **Nitrogen Dioxide Models** | **Degrees of freedom** | **AIC** |
| no2 | 23.70 | 509.44 |
| no27 | 22.28 | 508.09 |
| no215 | 25.34 | 504.95 |
| no230 | 26.57 | 505.28 |
| no260 | 26.19 | 502.09 |
| no290 | 24.91 | 504.33 |
| no2120 | 24.15 | 507.04 |
| no2180 | 24.35 | 506.90 |
| no2270 | 22.44 | 506.32 |
| no2365 | 22.57 | 500.22 |
| **Ozone Models** | **Degrees of freedom** | **AIC** |
| o3 | 22.49 | 509.71 |
| o37 | 22.52 | 508.92 |
| o315 | 26.31 | 506.33 |
| o330 | 25.75 | 504.55 |
| o360 | 22.54 | 509.54 |
| o390 | 22.62 | 507.79 |
| o3120 | 23.90 | 506.06 |
| o3180 | 22.57 | 494.99 |
| o3270 | 22.62 | 481.95 |
| o3365 | 24.23 | 489.36 |

**Table S3.** Selected Moving Average Pollution and Weather values for top model selection

| **Variables** | **Mean (Min-Max)** | **Median (Q1-Q3)** |
| --- | --- | --- |
| **Wind speed (mph):** 365 day MAv | 8.19 (7.79-9.09) | 7.96 (7.88-8.56) |
| **Temperature (°C):** 180 day MAv | 13.17 (8.07-17.77) | 13.26 (10.25-16.37) |
| **Relative humidity (%):** 180 day MAv | 71.62 (66.31-78.56) | 70.59 (68.43-74.97) |
| **PM_10_ (µg/m^3^):** 270 day MAv | 22.47 (18.41-27.67) | 21.10 (20.59-24.59) |
| **PM_2.5_ (µg/m^3^):** 120 day MAv | 13.11 (10.08-16.94) | 13.04 (11.07-15.01) |
| **Nitric oxide (µg/m^3^):** 365 day MAv | 43.52 (34.77-48.69) | 43.08 (42.35-46.05) |
| **Nitrogen dioxide (µg/m^3^):** 365 day MAv | 50.75 (45.07-53.97) | 51.16 (49.37-52.67) |
| **Tropospheric ozone (µg/m^3^):** 270 day MAv | 26.44 (21.21-31.64) | 26.36 (24.25-28.88) |
| Min-Max, minimum and maximum range; Q1-Q3, 25th and 75th percentiles; MAv, moving average; PM_10_, fine particles with diameters of 10 micrometres or less, PM_2.5_, fine particles with diameters of 2.5 micrometres or less | | |

**Table S4.** Model output from models 2-9 in top set (A-H)

| **Table S4 A.** Top model (2): Linear parameter odds ratios versus EASI>10 | | | | | | | |
| --- | --- | --- | --- | --- | --- | --- | --- |
|  |  | | **R^2^_Adj_** | | **AIC** | |  |
|  |  | | 0.365 | | 478.73 | |  |
|  |  | | **C-Index** | | **Ncases** | |  |
|  |  | | 0.86 | | 579 | |  |
| **Linear Parameters:** | | | | | | |  |
|  | **OR** | | **95% CI** | | **p-value** | |  |
| **Season: Autumn (Ref)** | - | | - | |  | |  |
| **Season: Spring** | 3.75 | | 0.87-16.2 | | 0.076 | |  |
| **Season: Summer** | 3.34 | | 1.20-9.27 | | 0.021 | |  |
| **Season: Winter** | 3.82 | | 1.43-10.18 | | 0.007 | |  |
| **Female (Ref)** | - | | - | |  | |  |
| **Male** | 1.53 | | 0.92-2.55 | | 0.101 | |  |
| **Investigator 1 (Ref)** | - | | - | |  | |  |
| **Investigator 2** | 1.87 | | 0.93-3.78 | | 0.080 | |  |
| **Investigator 3** | 0.29 | | 0.07-1.29 | | 0.104 | |  |
| **Investigator 4** | 1.11 | | 0.39-3.18 | | 0.850 | |  |
| **ESeC class 1-3 (Ref)** | - | | - | |  | |  |
| **ESeC class 4-6** | 2.81 | | 1.42-5.58 | | 0.003 | |  |
| **ESeC class 7-9** | 1.39 | | 0.76-2.52 | | 0.282 | |  |
|  | | | | | | |  |
| *R*^2^_Adj_ , Adjusted *R*^2^*;* AIC, Akaike Information Criterion; C-Index; Concordance statistic; Ncases, Number of cases in model; OR, Odds ratio; 95% CI, 95% confidence interval; ESeC, European Socio-economic Classification | | | | | | |  |
|  |  |  | |  | |  |  |

| **Table S4 B.** Top model (3): Linear parameter odds ratios versus EASI>10 | | | | | |
| --- | --- | --- | --- | --- | --- |
|  |  | | **R^2^_Adj_** | | **AIC** |
|  |  | | 0.360 | | 479.56 |
|  |  | | **C-Index** | | **Ncases** |
|  |  | | 0.87 | | 579 |
| **Linear Parameters:** | | | | | |
|  | **OR** | | **95% CI** | | **p-value** |
| **Season: Autumn (Ref)** | - | | - | |  |
| **Season: Spring** | 4.05 | | 0.92-17.76 | | 0.063 |
| **Season: Summer** | 3.44 | | 1.23-9.59 | | 0.018 |
| **Season: Winter** | 4.07 | | 1.51-10.97 | | 0.006 |
| **Investigator 1 (Ref)** | - | | - | |  |
| **Investigator 2** | 2.08 | | 1.04-4.16 | | 0.038 |
| **Investigator 3** | 0.30 | | 0.07-1.30 | | 0.106 |
| **Investigator 4** | 1.07 | | 0.37-3.14 | | 0.897 |
| **ESeC class 1-3 (Ref)** | - | | - | |  |
| **ESeC class 4-6** | 2.85 | | 1.43-5.67 | | 0.003 |
| **ESeC class 7-9** | 1.47 | | 0.81-2.68 | | 0.209 |
|  | | | | | |
| *R*^2^_Adj_ , Adjusted *R*^2^*;* AIC, Akaike Information Criterion; C-Index; Concordance statistic; Ncases, Number of cases in model; OR, Odds ratio; 95% CI, 95% confidence interval; ESeC, European Socio-economic Classification | | | | | |
|  |  |  | |  |  |

| **Table S4 C.** Top model (4): Linear parameter odds ratios versus EASI>10 | | | | | | | |  |
| --- | --- | --- | --- | --- | --- | --- | --- | --- |
|  |  | | **R^2^_Adj_** | | | **AIC** | |  |
|  |  | | 0.346 | | | 480.39 | |  |
|  |  | | **C-Index** | | | **Ncases** | |  |
|  |  | | 0.86 | | | 579 | |  |
| **Linear Parameters:** | | | | | | | |  |
|  | | **OR** | **95% CI** | | | **p-value** | |  |
| **Season: Autumn (Ref)** | | - | - | | |  | |  |
| **Season: Spring** | | 4.41 | 1.52-12.81 | | | 0.006 | |  |
| **Season: Summer** | | 3.49 | 1.73-7.04 | | | <0.001 | |  |
| **Season: Winter** | | 4.31 | 1.94-9.6 | | | <0.001 | |  |
| **Female (Ref)** | | - | - | | |  | |  |
| **Male** | | 1.56 | 0.94-2.59 | | | 0.084 | |  |
| **Investigator 1 (Ref)** | | - | - | | |  | |  |
| **Investigator 2** | | 2.05 | 1.06-3.99 | | | 0.035 | |  |
| **Investigator 3** | | 0.34 | 0.08-1.43 | | | 0.139 | |  |
| **Investigator 4** | | 1.37 | 0.48-3.94 | | | 0.555 | |  |
| **ESeC class 1-3 (Ref)** | | - | - | | |  | |  |
| **ESeC class 4-6** | | 2.77 | 1.4-5.49 | | | 0.003 | |  |
| **ESeC class 7-9** | | 1.33 | 0.73-2.42 | | | 0.347 | |  |
|  | | | | | | | |  |
| *R*^2^_Adj_ , Adjusted *R*^2^*;* AIC, Akaike Information Criterion; C-Index; Concordance statistic; Ncases, Number of cases in model; OR, Odds ratio; 95% CI, 95% confidence interval; ESeC, European Socio-economic Classification | | | | | | | |  |
|  |  | | |  |  | |  | |

| **Table S4 D.** Top model (5): Linear parameter odds ratios versus EASI>10 | | | | | | |
| --- | --- | --- | --- | --- | --- | --- |
|  |  | | | **R^2^_Adj_** | | **AIC** |
|  |  | | | 0.351 | | 480.92 |
|  |  | | | **C-Index** | | **Ncases** |
|  |  | | | 0.86 | | 579 |
| **Linear Parameters:** | | | | | | |
|  | | **OR** | | **95% CI** | | **p-value** |
| **Female (Ref)** | | - | | - | |  |
| **Male** | | 1.58 | | 0.95-2.62 | | 0.079 |
| **Investigator 1 (Ref)** | | - | | - | |  |
| **Investigator 2** | | 1.74 | | 0.88-3.43 | | 0.109 |
| **Investigator 3** | | 0.31 | | 0.07-1.4 | | 0.128 |
| **Investigator 4** | | 1.1 | | 0.38-3.18 | | 0.855 |
| **ESeC class 1-3 (Ref)** | | - | | - | |  |
| **ESeC class 4-6** | | 2.57 | | 1.31-5.04 | | 0.006 |
| **ESeC class 7-9** | | 1.41 | | 0.78-2.57 | | 0.257 |
|  | | | | | | |
| *R*^2^_Adj_ , Adjusted *R*^2^*;* AIC, Akaike Information Criterion; C-Index; Concordance statistic; Ncases, Number of cases in model; OR, Odds ratio; 95% CI, 95% confidence interval; ESeC, European Socio-economic Classification | | | | | | |
|  |  | |  | |  |  |

| **Table S4 E.** Top model (6): Linear parameter odds ratios versus EASI>10 | | | | | | |
| --- | --- | --- | --- | --- | --- | --- |
|  |  | | | **R^2^_Adj_** | | **AIC** |
|  |  | | | 0.363 | | 482.53 |
|  |  | | | **C-Index** | | **Ncases** |
|  |  | | | 0.87 | | 579 |
| **Linear Parameters:** | | | | | | |
|  | | **OR** | | **95% CI** | | **p-value** |
| **Season: Autumn (Ref)** | | - | | - | |  |
| **Season: Spring** | | 4.2 | | 0.97-18.12 | | 0.054 |
| **Season: Summer** | | 3.96 | | 1.31-11.99 | | 0.014 |
| **Season: Winter** | | 4.92 | | 1.64-14.78 | | 0.004 |
| **Female (Ref)** | | - | | - | |  |
| **Male** | | 1.89 | | 1.15-3.11 | | 0.012 |
| **ESeC class 1-3 (Ref)** | | - | | - | |  |
| **ESeC class 4-6** | | 2.77 | | 1.4-5.49 | | 0.003 |
| **ESeC class 7-9** | | 1.4 | | 0.77-2.54 | | 0.274 |
|  | | | | | | |
| *R*^2^_Adj_ , Adjusted *R*^2^*;* AIC, Akaike Information Criterion; C-Index; Concordance statistic; Ncases, Number of cases in model; OR, Odds ratio; 95% CI, 95% confidence interval; ESeC, European Socio-economic Classification | | | | | | |
|  |  | |  | |  |  |

| **Table S4 F.** Top model (7): Linear parameter odds ratios versus EASI>10 | | | | | | |
| --- | --- | --- | --- | --- | --- | --- |
|  |  | | | **R^2^_Adj_** | | **AIC** |
|  |  | | | 0.363 | | 482.55 |
|  |  | | | **C-Index** | | **Ncases** |
|  |  | | | 0.86 | | 579 |
| **Linear Parameters:** | | | | | | |
|  | | **OR** | | **95% CI** | | **p-value** |
| **Season: Autumn (Ref)** | | - | | - | |  |
| **Season: Spring** | | 3.83 | | 0.89-16.44 | | 0.070 |
| **Season: Summer** | | 2.8 | | 1.04-7.56 | | 0.042 |
| **Season: Winter** | | 3.56 | | 1.34-9.46 | | 0.011 |
| **Female (Ref)** | | - | | - | |  |
| **Male** | | 1.86 | | 1.13-3.05 | | 0.014 |
| **ESeC class 1-3 (Ref)** | | - | | - | |  |
| **ESeC class 4-6** | | 2.75 | | 1.39-5.44 | | 0.004 |
| **ESeC class 7-9** | | 1.33 | | 0.73-2.4 | | 0.346 |
|  | | | | | | |
| *R*^2^_Adj_ , Adjusted *R*^2^*;* AIC, Akaike Information Criterion; C-Index; Concordance statistic; Ncases, Number of cases in model; OR, Odds ratio; 95% CI, 95% confidence interval; ESeC, European Socio-economic Classification | | | | | | |
|  |  | |  | |  |  |

| **Table S4 G.** Top model (8): Linear parameter odds ratios versus EASI>10 | | | | | | |
| --- | --- | --- | --- | --- | --- | --- |
|  |  | | | **R^2^_Adj_** | | **AIC** |
|  |  | | | 0.365 | | 483.16 |
|  |  | | | **C-Index** | | **Ncases** |
|  |  | | | 0.87 | | 579 |
| **Linear Parameters:** | | | | | | |
|  | | **OR** | | **95% CI** | | **p-value** |
| **Season: Autumn (Ref)** | | - | | - | |  |
| **Season: Spring** | | 3.46 | | 0.8-14.97 | | 0.096 |
| **Season: Summer** | | 2.75 | | 1.02-7.45 | | 0.045 |
| **Season: Winter** | | 3.8 | | 1.42-10.2 | | 0.008 |
| **Female (Ref)** | | - | | - | |  |
| **Male** | | 1.63 | | 0.98-2.7 | | 0.061 |
| **Investigator 1 (Ref)** | | - | | - | |  |
| **Investigator 2** | | 2.03 | | 1.01-4.1 | | 0.047 |
| **Investigator 3** | | 0.34 | | 0.08-1.5 | | 0.154 |
| **Investigator 4** | | 1.3 | | 0.45-3.76 | | 0.622 |
|  | | | | | | |
| *R*^2^_Adj_ , Adjusted *R*^2^*;* AIC, Akaike Information Criterion; C-Index; Concordance statistic; Ncases, Number of cases in model; OR, Odds ratio; 95% CI, 95% confidence interval; ESeC, European Socio-economic Classification | | | | | | |
|  |  | |  | |  |  |

| **Table S4 H.** Top model (9): Linear parameter odds ratios versus EASI>10 | | | | | | | | |
| --- | --- | --- | --- | --- | --- | --- | --- | --- |
|  |  | | | | **R^2^_Adj_** | | **AIC** | |
|  |  | | | | 0.360 | | 483.54 | |
|  |  | | | | **C-Index** | | **Ncases** | |
|  |  | | | | 0.87 | | 579 | |
| **Linear Parameters:** | | | | | | | | |
|  | | **OR** | | **95% CI** | | | **p-value** |  |
| **Season: Autumn (Ref)** | | - | | - | | |  |  |
| **Season: Spring** | | 3.63 | | 0.77-17.08 | | | 0.102 |  |
| **Season: Summer** | | 3.4 | | 1.02-11.3 | | | 0.045 |  |
| **Season: Winter** | | 8.5 | | 2.15-33.55 | | | 0.002 |  |
| **Female (Ref)** | | - | | - | | |  |  |
| **Male** | | 1.66 | | 0.99-2.78 | | | 0.055 |  |
| **Investigator 1 (Ref)** | | - | | - | | |  |  |
| **Investigator 2** | | 1.84 | | 0.88-3.86 | | | 0.105 |  |
| **Investigator 3** | | 0.32 | | 0.07-1.44 | | | 0.136 |  |
| **Investigator 4** | | 1.14 | | 0.39-3.33 | | | 0.812 |  |
| **ESeC class 1-3 (Ref)** | | - | | - | | |  |  |
| **ESeC class 4-6** | | 2.88 | | 1.43-5.76 | | | 0.003 |  |
| **ESeC class 7-9** | | 1.45 | | 0.79-2.67 | | | 0.229 |  |
|  | | | | | | | | |
| *R*^2^_Adj_ , Adjusted *R*^2^*;* AIC, Akaike Information Criterion; C-Index; Concordance statistic;Ncases, Number of cases in model; OR, Odds ratio; 95% CI, 95% confidence interval; ESeC, European Socio-economic Classification | | | | | | | | |
|  |  | |  | | |  |  | |

**Table S5.** Initial moving average model AIC values in Korean dataset

| **<10µm Particulate Matter Models** | **Degrees of freedom** | **AIC** |
| --- | --- | --- |
| pm10 | 7.00 | 249.70 |
| pm107 | 7.00 | 249.99 |
| pm1015 | 11.05 | 248.10 |
| pm1030 | 7.00 | 238.98 |
| pm1060 | 8.94 | 224.14 |
| pm1090 | 7.00 | 235.90 |
| pm10120 | 10.65 | 229.76 |
| pm10180 | 7.97 | 236.31 |
| pm10270 | 11.69 | 237.39 |
| pm10365 | 7.00 | 237.63 |
| **Nitrogen Dioxide Models** | **Degrees of freedom** | **AIC** |
| no2 | 7.00 | 250.21 |
| no27 | 9.05 | 245.30 |
| no215 | 11.76 | 237.54 |
| no230 | 10.66 | 238.92 |
| no260 | 10.65 | 239.99 |
| no290 | 11.25 | 232.57 |
| no2120 | 7.00 | 236.17 |
| no2180 | 10.95 | 234.74 |
| no2270 | 9.76 | 233.30 |
| no2365 | 12.17 | 232.20 |
| **Ozone Models** | **Degrees of freedom** | **AIC** |
| o3 | 9.19 | 247.66 |
| o37 | 8.24 | 250.24 |
| o315 | 8.34 | 248.01 |
| o330 | 9.10 | 245.54 |
| o360 | 11.70 | 238.95 |
| o390 | 12.03 | 241.11 |
| o3120 | 7.00 | 249.74 |
| o3180 | 12.07 | 238.69 |
| o3270 | 11.30 | 234.57 |
| o3365 | 7.00 | 239.44 |
| o3365 | 7.00 | 239.44 |
